# Bayesian models for pooling microarray studies with multiple sources of replications

**DOI:** 10.1186/1471-2105-7-247

**Published:** 2006-05-05

**Authors:** Erin M Conlon, Joon J Song, Jun S Liu

**Affiliations:** 1 Department of Mathematics and Statistics, University of Massachusetts, Amherst, Massachusetts, USA; 2 Department of Statistics, Harvard University, Cambridge, Massachusetts, USA

## Abstract

**Background:**

Biologists often conduct multiple but different cDNA microarray studies that all target the same biological system or pathway. Within each study, replicate slides within repeated identical experiments are often produced. Pooling information across studies can help more accurately identify true target genes. Here, we introduce a method to integrate multiple independent studies efficiently.

**Results:**

We introduce a Bayesian hierarchical model to pool cDNA microarray data across multiple independent studies to identify highly expressed genes. Each study has multiple sources of variation, i.e. replicate slides within repeated identical experiments. Our model produces the gene-specific posterior probability of differential expression, which provides a direct method for ranking genes, and provides Bayesian estimates of false discovery rates (FDR). In simulations combining two and five independent studies, with fixed FDR levels, we observed large increases in the number of discovered genes in pooled versus individual analyses. When the number of output genes is fixed (e.g., top 100), the pooled model found appreciably more truly differentially expressed genes than the individual studies. We were also able to identify more differentially expressed genes from pooling two independent studies in *Bacillus subtilis *than from each individual data set. Finally, we observed that in our simulation studies our Bayesian FDR estimates tracked the true FDRs very well.

**Conclusion:**

Our method provides a cohesive framework for combining multiple but not identical microarray studies with several sources of replication, with data produced from the same platform. We assume that each study contains only two conditions: an experimental and a control sample. We demonstrated our model's suitability for a small number of studies that have been either pre-scaled or have no outliers.

## Background

cDNA microarrays monitor gene expression for thousands of genes simultaneously. Two experimental conditions are compared by examining the ratio of expression between two samples, e.g. treatment versus control, wildtype versus mutant, or disease versus healthy. The primary goal of these experiments is to identify genes that are differentially expressed between the two conditions. The up-regulated and down-regulated genes shed light on biological mechanisms of the cell, such as functional pathways, response to treatments, and gene regulation.

Bayesian models have been used extensively in single microarray studies to identify differentially expressed genes (e.g. [1-3]); the discrete mixture model approach in particular has had considerable use [4-13]. Additional Bayesian methods for identifying differentially expressed genes include Bayesian ANOVA for microarrays (BAM) of Ishwaran and Rao [14,15]. This approach redefines the search for differentially expressed genes as a Bayesian variable selection process and uses a hierarchical model that is tailored to adaptive shrinkage. Through the use of model averaging, BAM essentially shrinks only the effects of the non-differentially expressed genes relative to the least squares estimates. For a review of Bayesian approaches to microarray data analysis and their advantages over frequentist methods, see Yang *et al. *[16].

In addition to performing single microarray studies, biologists often conduct multiple but not identical studies to understand the same biological system. Pooling results of these studies can help identify truly differentially expressed genes. Meta-analyses for microarray studies have been used recently by many researchers in a non-Bayesian context [17-24]. Rhodes *et al. *[17] combined the results of four prostate cancer studies using a *p*-value approach. Genes were assigned a *p*-value in each study separately, and the results were combined to estimate a gene-specific *p*-value across all studies. This method avoids the necessity of integrating gene expression measures and thus can be used for data across multiple platforms. Choi *et al. *[18] presented a meta-analysis that integrated gene effect sizes, rather than *p*-values, into one mean effect. The effect sizes for each study were equal to the mean differences between affected and control groups, standardized by a pooled standard deviation. Due to this standardization, data was able to be integrated across platforms. A common parameter for inter-study variability was incorporated into the model, and statistical significance was determined by permutation tests. Parmigiani *et al. *[20] introduced an integrative correlation approach to combining data from multiple platforms. This procedure evaluated gene expression consistencies across platforms rather than pooling gene expression values. Using lung cancer data, this method identified genes with reproducible expression patterns across studies and improved correlation across studies. Additional theoretical approaches for combining data from different platforms include adding covariates to models to account for the differences among data types [24,25], although this has not been applied in a microarray setting. While the studies of Rhodes *et al. *[17], Choi *et al. *[18], Parmigiani *et al. *[20] and others provide methods for integrating data across platforms, other authors have shown the difficulties in such an approach ([26,27]; discussions in [25,28]). Working with cell lines, Kuo *et al. *[26] and Jarvinen *et al. *[27] both conclude that combining data across platforms is unreliable. Due to these difficulties, other meta-analysis methods focus on incorporating data from one platform only [23,24]. Here, we focus our approach to combine microarray data from the same platform, cDNA microarrays, and assume that the data has either been pre-normalized across studies or that there are no outlying studies.

Choi *et al. *[18] also provided an alternative Bayesian meta-analysis method to their random effects approach. In this Bayesian model, uninformative prior distributions were assigned to the overall mean effects and the inter-study variation parameter. Within-study gene effects were modelled as *t*-distributions, and posterior estimates of the overall mean effect for each gene were produced by smoothing effects across studies. The authors demonstrated that Bayesian meta-analysis is more robust and flexible than traditional methods, confirming the findings of DuMouchel and Harris [29]. Bayesian models are also well-suited to data with many levels of replication, including replicate slides within repeated identical experiments. Due to these advantages, we introduce a Bayesian hierarchical model that provides a principled framework for incorporating data from multiple independent cDNA microarray studies with several sources of replication. Unlike the approach of Choi *et al. *[18], which smoothes the gene effects into one average, our method produces the posterior probability of differential expression based on gene expression levels across studies. Thus, inter-study variability does not need to be estimated by our model. The probability of differential expression provides a direct method for ranking genes, and also for estimating both integration-driven discovery rates and false discovery rates. In simulations, we illustrate that pooling studies increases the number of discovered genes for given thresholds of probabilities of differential expression and false discovery rates, compared to individual studies. In addition, for a fixed top number of genes, the pooled model identifies considerably more differentially expressed genes than separate studies. We also illustrate our method using experimental data from two independent studies in *Bacillus (B.) subtilis*.

### Background on cDNA microarray experiments

cDNA microarrays measure the amount of messenger RNA (mRNA) contained in an experimental sample. They are produced by robotic arrayers, which place entire gene sequences complementary to mRNA onto glass slides. In an experiment, the mRNA in two samples, e.g. treated and control, are fluorescently labeled with two different dyes, typically red and green (Cy5 and Cy3), and mixed together. The combined sample is hybridized to the array, and complementary sequences bind to each other. The relative amounts of mRNA present in the two samples are measured by scanning the slide with two different wavelengths. The resulting fluorescent intensity values for the red and green-labeled mRNA are then compared by using the ratio of intensities. For further details, see [30-33]. We use log-ratios of intensities in each study since they even out highly skewed distributions and give a more realistic sense of variation [34].

## Results and discussion

### Bayesian model for pooling multiple studies

Biologists often conduct multiple but different studies that all target the same biological system or pathway. For example, when studying the effect of a key transcription factor *σ*^E ^in *B. subtilis*, Eichenberger *et al. *[35] conducted both the *σ*^E ^knockout and the *σ*^E ^over-expression experiments (i.e. the mutant and induction experiments, respectively; see Methods). Thus, those genes that are up-regulated in one experiment should be down-regulated in another. Pooling both experiments can help more accurately identify true target genes. More generally, we may imagine having available multiple independent studies of one specific biological system. We assume that each study contains only two conditions: an experimental and a control. It is desirable to combine information from these studies in a principled way. Our model to achieve this goal is as follows:

yjgse|μjge~N(μjge,τjg2), j=1,…,J;g=1,…,G;e=1,…,E;s=1,…,Seμjge|θjg~N(θjg,σjg2), j=1,…,J;g=1,…,G;e=1,…,Eθjg|Ig=0~N(0,ηjg02)θjg|Ig=1~N(0,cj×ηjg02)Ig~Bernoulli(p)p~Uniform(0,1),     (1)
 MathType@MTEF@5@5@+=feaafiart1ev1aaatCvAUfKttLearuWrP9MDH5MBPbIqV92AaeXatLxBI9gBamXvP5wqSXMqHnxAJn0BKvguHDwzZbqegyvzYrwyUfgarqqtubsr4rNCHbGeaGqiA8vkIkVAFgIELiFeLkFeLk=iY=Hhbbf9v8qqaqFr0xc9pk0xbba9q8WqFfeaY=biLkVcLq=JHqVepeea0=as0db9vqpepesP0xe9Fve9Fve9GapdbaqaaeGacaGaaiaabeqaamqadiabaaGcbaqcaaAbaeaabyWaaaaabiqaaGhacaWLjaGaemyEaKNcdaWgaaqcbaAaaiabdQgaQjabdEgaNjabdohaZjabdwgaLbqabaqcaaQaeiiFaWhcciGae8hVd0McdaWgaaqcbaAaaiabdQgaQjabdEgaNjabdwgaLbqabaaajaaObaGaeiOFa4habaGaeeOta4KcdaqadaqcaaAaaiab=X7aTPWaaSbaaKqaGgaacqWGQbGAcqWGNbWzcqWGLbqzaeqaaKaaGkabcYcaSiab=r8a0PWaa0baaKqaGgaacqWGQbGAcqWGNbWzaeaacqaIYaGmaaaajaaOcaGLOaGaayzkaaGaeiilaWIaemOAaOMaeyypa0JaeGymaeJaeiilaWIaeSOjGSKaeiilaWIaemOsaOKaei4oaSJaem4zaCMaeyypa0JaeGymaeJaeiilaWIaeSOjGSKaeiilaWIaem4raCKaei4oaSJaemyzauMaeyypa0JaeGymaeJaeiilaWIaeSOjGSKaeiilaWIaemyrauKaei4oaSJaem4CamNaeyypa0JaeGymaeJaeiilaWIaeSOjGSKaeiilaWIaem4uamLcdaWgaaqcbaAaaiabdwgaLbqabaaajaaObiqaaqracaWLjaGae8hVd0McdaWgaaqcbaAaaiabdQgaQjabdEgaNjabdwgaLbqabaqcaaQaeiiFaWNae8hUdeNcdaWgaaqcbaAaaiabdQgaQjabdEgaNbqabaaajaaObaGaeiOFa4habaGaeeOta4KcdaqadaqcaaAaaiab=H7aXPWaaSbaaKqaGgaacqWGQbGAcqWGNbWzaeqaaKaaGkabcYcaSiab=n8aZPWaa0baaKqaGgaacqWGQbGAcqWGNbWzaeaacqaIYaGmaaaajaaOcaGLOaGaayzkaaGaeiilaWIaemOAaOMaeyypa0JaeGymaeJaeiilaWIaeSOjGSKaeiilaWIaemOsaOKaei4oaSJaem4zaCMaeyypa0JaeGymaeJaeiilaWIaeSOjGSKaeiilaWIaem4raCKaei4oaSJaemyzauMaeyypa0JaeGymaeJaeiilaWIaeSOjGSKaeiilaWIaemyraueabaGae8hUdeNcdaWgaaqcbaAaaiabdQgaQjabdEgaNbqabaqcaaQaeiiFaWNaemysaKKcdaWgaaqcbaAaaiabdEgaNbqabaqcaaQaeyypa0JaeGimaadabaGaeiOFa4habaGaeeOta4KcdaqadaqcaaAaaiabicdaWiabcYcaSiab=D7aOPWaa0baaKqaGgaacqWGQbGAcqWGNbWzcqaIWaamaeaacqaIYaGmaaaajaaOcaGLOaGaayzkaaaabaGae8hUdeNcdaWgaaqcbaAaaiabdQgaQjabdEgaNbqabaqcaaQaeiiFaWNaemysaKKcdaWgaaqcbaAaaiabdEgaNbqabaqcaaQaeyypa0JaeGymaedabaGaeiOFa4habaGaeeOta4KcdaqadaqcaaAaaiabicdaWiabcYcaSiabdogaJPWaaSbaaKqaGgaacqWGQbGAaeqaaKaaGkabgEna0kab=D7aOPWaa0baaKqaGgaacqWGQbGAcqWGNbWzcqaIWaamaeaacqaIYaGmaaaajaaOcaGLOaGaayzkaaaabiqaaWfbcaWLjaGaemysaKKcdaWgaaqcbaAaaiabdEgaNbqabaaajaaObaGaeiOFa4habaGaeeOqaiKaeeyzauMaeeOCaiNaeeOBa4Maee4Ba8MaeeyDauNaeeiBaWMaeeiBaWMaeeyAaKMcdaqadaqcaaAaaiabdchaWbGaayjkaiaawMcaaaqaaiaaxMaacqWGWbaCaeaacqGG+bGFaeaacqqGvbqvcqqGUbGBcqqGPbqAcqqGMbGzcqqGVbWBcqqGYbGCcqqGTbqBkmaabmaajaaObaGaeGimaaJaeiilaWIaeGymaedacaGLOaGaayzkaaGaeiilaWcaaiaaxMaacaWLjaGcdaqadaqcaaAaaiabigdaXaGaayjkaiaawMcaaaaa@2D8D@

for *j *= 1,..., *J *independent studies. Here, *y*_*jgse *_is the microarray data, i.e. the normalized log-expression ratios for gene *g*, experiment *e*, slide *s*, and *μ*_*jge *_is the average over all slides *S*_*e *_within experiment *e *of study *j*. *θ*_*jg *_is the log-expression ratio for each gene of study *j*. Conjugate inverse chi-squared prior distributions are assigned to τjg2
 MathType@MTEF@5@5@+=feaafiart1ev1aaatCvAUfKttLearuWrP9MDH5MBPbIqV92AaeXatLxBI9gBaebbnrfifHhDYfgasaacH8akY=wiFfYdH8Gipec8Eeeu0xXdbba9frFj0=OqFfea0dXdd9vqai=hGuQ8kuc9pgc9s8qqaq=dirpe0xb9q8qiLsFr0=vr0=vr0dc8meaabaqaciaacaGaaeqabaqabeGadaaakeaaiiGacqWFepaDdaqhaaWcbaGaemOAaOMaem4zaCgabaGaeGOmaidaaaaa@324B@ and σjg2
 MathType@MTEF@5@5@+=feaafiart1ev1aaatCvAUfKttLearuWrP9MDH5MBPbIqV92AaeXatLxBI9gBaebbnrfifHhDYfgasaacH8akY=wiFfYdH8Gipec8Eeeu0xXdbba9frFj0=OqFfea0dXdd9vqai=hGuQ8kuc9pgc9s8qqaq=dirpe0xb9q8qiLsFr0=vr0=vr0dc8meaabaqaciaacaGaaeqabaqabeGadaaakeaaiiGacqWFdpWCdaqhaaWcbaGaemOAaOMaem4zaCgabaGaeGOmaidaaaaa@3249@ , for which we use the notation τjg2~kτ˜j2/χk2
 MathType@MTEF@5@5@+=feaafiart1ev1aaatCvAUfKttLearuWrP9MDH5MBPbIqV92AaeXatLxBI9gBaebbnrfifHhDYfgasaacH8akY=wiFfYdH8Gipec8Eeeu0xXdbba9frFj0=OqFfea0dXdd9vqai=hGuQ8kuc9pgc9s8qqaq=dirpe0xb9q8qiLsFr0=vr0=vr0dc8meaabaqaciaacaGaaeqabaqabeGadaaakeaaiiGacqWFepaDdaqhaaWcbaGaemOAaOMaem4zaCgabaGaeGOmaidaaOGaeiOFa4Naem4AaSMaf8hXdqNbaGaadaqhaaWcbaGaemOAaOgabaGaeGOmaidaaOGaei4la8Iae83Xdm2aa0baaSqaaiabdUgaRbqaaiabikdaYaaaaaa@3EA3@ and σjg2~hσ˜j2/χh2
 MathType@MTEF@5@5@+=feaafiart1ev1aaatCvAUfKttLearuWrP9MDH5MBPbIqV92AaeXatLxBI9gBaebbnrfifHhDYfgasaacH8akY=wiFfYdH8Gipec8Eeeu0xXdbba9frFj0=OqFfea0dXdd9vqai=hGuQ8kuc9pgc9s8qqaq=dirpe0xb9q8qiLsFr0=vr0=vr0dc8meaabaqaciaacaGaaeqabaqabeGadaaakeaaiiGacqWFdpWCdaqhaaWcbaGaemOAaOMaem4zaCgabaGaeGOmaidaaOGaeiOFa4NaemiAaGMaf83WdmNbaGaadaqhaaWcbaGaemOAaOgabaGaeGOmaidaaOGaei4la8Iae83Xdm2aa0baaSqaaiabdIgaObqaaiabikdaYaaaaaa@3E93@. Here, (χk2)−1
 MathType@MTEF@5@5@+=feaafiart1ev1aaatCvAUfKttLearuWrP9MDH5MBPbIqV92AaeXatLxBI9gBaebbnrfifHhDYfgasaacH8akY=wiFfYdH8Gipec8Eeeu0xXdbba9frFj0=OqFfea0dXdd9vqai=hGuQ8kuc9pgc9s8qqaq=dirpe0xb9q8qiLsFr0=vr0=vr0dc8meaabaqaciaacaGaaeqabaqabeGadaaakeaacqGGOaakiiGacqWFhpWydaqhaaWcbaGaem4AaSgabaGaeGOmaidaaOGaeiykaKYaaWbaaSqabeaacqGHsislcqaIXaqmaaaaaa@34AE@ denotes the standard inverse *χ*^2 ^distribution with *k *degrees of freedom, and τ˜j2
 MathType@MTEF@5@5@+=feaafiart1ev1aaatCvAUfKttLearuWrP9MDH5MBPbIqV92AaeXatLxBI9gBaebbnrfifHhDYfgasaacH8akY=wiFfYdH8Gipec8Eeeu0xXdbba9frFj0=OqFfea0dXdd9vqai=hGuQ8kuc9pgc9s8qqaq=dirpe0xb9q8qiLsFr0=vr0=vr0dc8meaabaqaciaacaGaaeqabaqabeGadaaakeaaiiGacuWFepaDgaacamaaDaaaleaacqWGQbGAaeaacqaIYaGmaaaaaa@3103@ and σ˜j2
 MathType@MTEF@5@5@+=feaafiart1ev1aaatCvAUfKttLearuWrP9MDH5MBPbIqV92AaeXatLxBI9gBaebbnrfifHhDYfgasaacH8akY=wiFfYdH8Gipec8Eeeu0xXdbba9frFj0=OqFfea0dXdd9vqai=hGuQ8kuc9pgc9s8qqaq=dirpe0xb9q8qiLsFr0=vr0=vr0dc8meaabaqaciaacaGaaeqabaqabeGadaaakeaaiiGacuWFdpWCgaacamaaDaaaleaacqWGQbGAaeaacqaIYaGmaaaaaa@3101@ are scale parameters of the inverse chi-squared distribution and are derived from the data. The parameter τ˜j2
 MathType@MTEF@5@5@+=feaafiart1ev1aaatCvAUfKttLearuWrP9MDH5MBPbIqV92AaeXatLxBI9gBaebbnrfifHhDYfgasaacH8akY=wiFfYdH8Gipec8Eeeu0xXdbba9frFj0=OqFfea0dXdd9vqai=hGuQ8kuc9pgc9s8qqaq=dirpe0xb9q8qiLsFr0=vr0=vr0dc8meaabaqaciaacaGaaeqabaqabeGadaaakeaaiiGacuWFepaDgaacamaaDaaaleaacqWGQbGAaeaacqaIYaGmaaaaaa@3103@ is equal to slide variation, σ˜j2
 MathType@MTEF@5@5@+=feaafiart1ev1aaatCvAUfKttLearuWrP9MDH5MBPbIqV92AaeXatLxBI9gBaebbnrfifHhDYfgasaacH8akY=wiFfYdH8Gipec8Eeeu0xXdbba9frFj0=OqFfea0dXdd9vqai=hGuQ8kuc9pgc9s8qqaq=dirpe0xb9q8qiLsFr0=vr0=vr0dc8meaabaqaciaacaGaaeqabaqabeGadaaakeaaiiGacuWFdpWCgaacamaaDaaaleaacqWGQbGAaeaacqaIYaGmaaaaaa@3101@ is equal to experiment variation for study *j*, and the degrees of freedom *h, k *are assumed known. We define *I*_*g*_~Bernoulli(*p*) as the indicator variable for differential expression of gene *g*, i.e. *θ*_*jg*_≠0, *j *= 1,..., *J*, where *p *is the percent of differentially expressed genes. Thus, Prob(*I*_*g *_= 1) = *p*, where

Ig={0 if θjg=0,j=1,…,J1 if θjg≠0,j=1,…,J
 MathType@MTEF@5@5@+=feaafiart1ev1aaatCvAUfKttLearuWrP9MDH5MBPbIqV92AaeXatLxBI9gBaebbnrfifHhDYfgasaacH8akY=wiFfYdH8Gipec8Eeeu0xXdbba9frFj0=OqFfea0dXdd9vqai=hGuQ8kuc9pgc9s8qqaq=dirpe0xb9q8qiLsFr0=vr0=vr0dc8meaabaqaciaacaGaaeqabaqabeGadaaakeaacqWGjbqsdaWgaaWcbaGaem4zaCgabeaakiabg2da9maaceaabaqbaeqabiGaaaqaaiabicdaWiabbccaGiabbMgaPjabbAgaMjabbccaGGGaciab=H7aXnaaBaaaleaacqWGQbGAcqWGNbWzaeqaaOGaeyypa0JaeGimaaJaeiilaWcabaGaemOAaOMaeyypa0JaeGymaeJaeiilaWIaeSOjGSKaeiilaWIaemOsaOeabaGaeGymaeJaeeiiaaIaeeyAaKMaeeOzayMaeeiiaaIae8hUde3aaSbaaSqaaiabdQgaQjabdEgaNbqabaGccqGHGjsUcqaIWaamcqGGSaalaeaacqWGQbGAcqGH9aqpcqaIXaqmcqGGSaalcqWIMaYscqGGSaalcqWGkbGsaaaacaGL7baaaaa@5A25@

Here, genes are divided into two groups, non-expressed (*I*_*g *_= 0) and expressed (*I*_*g *_= 1), with respective probabilities (1-*p*) and *p*. The model produces the posterior distribution for *D*_*g *_= Prob(*I*_*g *_= 1|data), which is the basis for inference. For prior distributions, when *I*_*g *_= 0, we assume the *θ*_*jg *_are distributed normally with mean zero and small variance ηjg02
 MathType@MTEF@5@5@+=feaafiart1ev1aaatCvAUfKttLearuWrP9MDH5MBPbIqV92AaeXatLxBI9gBaebbnrfifHhDYfgasaacH8akY=wiFfYdH8Gipec8Eeeu0xXdbba9frFj0=OqFfea0dXdd9vqai=hGuQ8kuc9pgc9s8qqaq=dirpe0xb9q8qiLsFr0=vr0=vr0dc8meaabaqaciaacaGaaeqabaqabeGadaaakeaaiiGacqWF3oaAdaqhaaWcbaGaemOAaOMaem4zaCMaeGimaadabaGaeGOmaidaaaaa@3320@; when *I*_*g *_= 1, we assume the *θ*_*jg *_are distributed normally with mean zero and large variance *c *× ηjg02
 MathType@MTEF@5@5@+=feaafiart1ev1aaatCvAUfKttLearuWrP9MDH5MBPbIqV92AaeXatLxBI9gBaebbnrfifHhDYfgasaacH8akY=wiFfYdH8Gipec8Eeeu0xXdbba9frFj0=OqFfea0dXdd9vqai=hGuQ8kuc9pgc9s8qqaq=dirpe0xb9q8qiLsFr0=vr0=vr0dc8meaabaqaciaacaGaaeqabaqabeGadaaakeaaiiGacqWF3oaAdaqhaaWcbaGaemOAaOMaem4zaCMaeGimaadabaGaeGOmaidaaaaa@3320@. A Markov chain Monte Carlo (MCMC) implementation of the model [36] simulates posterior distributions for each parameter. See Methods for more details on the prior distributions and the MCMC implementation. For each gene, we calculate the posterior probability *D*_*g *_of differential expression over all studies, and rank the genes based on *D*_*g*_. The prior estimates of the variance parameters τjg2
 MathType@MTEF@5@5@+=feaafiart1ev1aaatCvAUfKttLearuWrP9MDH5MBPbIqV92AaeXatLxBI9gBaebbnrfifHhDYfgasaacH8akY=wiFfYdH8Gipec8Eeeu0xXdbba9frFj0=OqFfea0dXdd9vqai=hGuQ8kuc9pgc9s8qqaq=dirpe0xb9q8qiLsFr0=vr0=vr0dc8meaabaqaciaacaGaaeqabaqabeGadaaakeaaiiGacqWFepaDdaqhaaWcbaGaemOAaOMaem4zaCgabaGaeGOmaidaaaaa@324B@ and σjg2
 MathType@MTEF@5@5@+=feaafiart1ev1aaatCvAUfKttLearuWrP9MDH5MBPbIqV92AaeXatLxBI9gBaebbnrfifHhDYfgasaacH8akY=wiFfYdH8Gipec8Eeeu0xXdbba9frFj0=OqFfea0dXdd9vqai=hGuQ8kuc9pgc9s8qqaq=dirpe0xb9q8qiLsFr0=vr0=vr0dc8meaabaqaciaacaGaaeqabaqabeGadaaakeaaiiGacqWFdpWCdaqhaaWcbaGaemOAaOMaem4zaCgabaGaeGOmaidaaaaa@3249@ are similar to Tseng *et al. *[2]. Our prior structure for a single experiment is similar to Gottardo *et al. *[8] except that we place a Uniform prior distribution on *p *rather than estimating *p *through an iterative algorithm. We also have one more level of variation than the model of Gottardo *et al. *[8], i.e. variation over slides within experiments. Our underlying hierarchical Gaussian model is also similar to the BAM models of Ishwaran and Rao [14,15], except that the BAM models are designed for a two-sample problem, while our model assumes that the data are ratios of treatment and control intensities. We evaluate our model using false discovery rates and integration-driven discovery rates, defined in the following.

### Integration-driven discovery

Choi *et al. *[18] define the integration-driven discovery rate (IDR) as the number of genes discovered in a meta-analysis that were not discovered in any of the individual studies alone, divided by the total number of discoveries. IDR represents the gain in information from combining studies versus individual studies. For our model, we fix a threshold value, *γ*, and label genes differentially expressed if (*D*_*g *_≥ *γ*). The IDR is defined as the number of genes that are labelled differentially expressed in the pooled analysis and are not differentially expressed in any of the individual studies:

IDR (γ)=# genes[(Dg≥γ) in pooled analysis] and [(Dg<γ) in all individual studies]# genes[(Dg≥γ) in pooled analysis].
 MathType@MTEF@5@5@+=feaafiart1ev1aaatCvAUfKttLearuWrP9MDH5MBPbIqV92AaeXatLxBI9gBaebbnrfifHhDYfgasaacH8akY=wiFfYdH8Gipec8Eeeu0xXdbba9frFj0=OqFfea0dXdd9vqai=hGuQ8kuc9pgc9s8qqaq=dirpe0xb9q8qiLsFr0=vr0=vr0dc8meaabaqaciaacaGaaeqabaqabeGadaaakeaacqqGjbqscqqGebarcqqGsbGucqqGGaaidaqadaqaaGGaciab=n7aNbGaayjkaiaawMcaaiabg2da9maalaaabaGaei4iamIaeeiiaaIaee4zaCMaeeyzauMaeeOBa4MaeeyzauMaee4CamNaee4waSLaeeikaGIaemiraq0aaSbaaSqaaiabdEgaNbqabaGccqGHLjYScqWFZoWzcqGGPaqkcqqGGaaicqqGPbqAcqqGUbGBcqqGGaaicqqGWbaCcqqGVbWBcqqGVbWBcqqGSbaBcqqGLbqzcqqGKbazcqqGGaaicqqGHbqycqqGUbGBcqqGHbqycqqGSbaBcqqG5bqEcqqGZbWCcqqGPbqAcqqGZbWCcqqGDbqxcqqGGaaicqqGHbqycqqGUbGBcqqGKbazcqqGGaaicqqGBbWwcqqGOaakcqWGebardaWgaaWcbaGaem4zaCgabeaakiabgYda8iab=n7aNjabcMcaPiabbccaGiabbMgaPjabb6gaUjabbccaGiabbggaHjabbYgaSjabbYgaSjabbccaGiabbMgaPjabb6gaUjabbsgaKjabbMgaPjabbAha2jabbMgaPjabbsgaKjabbwha1jabbggaHjabbYgaSjabbccaGiabbohaZjabbsha0jabbwha1jabbsgaKjabbMgaPjabbwgaLjabbohaZjabb2faDbqaaiabbocaJiabbccaGiabbEgaNjabbwgaLjabb6gaUjabbwgaLjabbohaZjabbUfaBjabbIcaOiabdseaenaaBaaaleaacqWGNbWzaeqaaOGaeyyzImRae83SdCMaeiykaKIaeeiiaaIaeeyAaKMaeeOBa4MaeeiiaaIaeeiCaaNaee4Ba8Maee4Ba8MaeeiBaWMaeeyzauMaeeizaqMaeeiiaaIaeeyyaeMaeeOBa4MaeeyyaeMaeeiBaWMaeeyEaKNaee4CamNaeeyAaKMaee4CamNaeeyxa0faaiabc6caUaaa@BBC0@

### False discovery rate

Benjamini and Hochberg [37] introduced the false discovery rate (FDR), which is defined as the number of false discoveries divided by the number of discoveries. We refer to this as the true false discovery rate (*t*FDR), which can be exactly computed in our simulation studies since we know which genes are truly differentially expressed. Further applications of FDR to microarrays include [38-40]. Genovese and Wasserman [41] define the posterior expected FDR (*pe*FDR) as:

peFDR=E(FDR|Y)=∑g(1−Dg)δg∑gδg,
 MathType@MTEF@5@5@+=feaafiart1ev1aaatCvAUfKttLearuWrP9MDH5MBPbIqV92AaeXatLxBI9gBamXvP5wqSXMqHnxAJn0BKvguHDwzZbqegyvzYrwyUfgarqqtubsr4rNCHbGeaGqiA8vkIkVAFgIELiFeLkFeLk=iY=Hhbbf9v8qqaqFr0xc9pk0xbba9q8WqFfeaY=biLkVcLq=JHqVepeea0=as0db9vqpepesP0xe9Fve9Fve9GapdbaqaaeGacaGaaiaabeqaamqadiabaaGcbaqcaaQaemiCaaNaemyzauMaeeOrayKaeeiraqKaeeOuaiLaeyypa0JaemyrauKcdaqadaqcaaAaaiabbAeagjabbseaejabbkfasjabcYha8HWadiaa=LfaaiaawIcacaGLPaaacqGH9aqpkmaalaaajaaObaGcdaaeqbqcaaAaaOWaaeWaaKaaGgaacqaIXaqmcqGHsislcqWGebarkmaaBaaajeaObaGaem4zaCgabeaaaKaaGkaawIcacaGLPaaaiiGacqGF0oazkmaaBaaajeaObaGaem4zaCgabeaaaeaacqWGNbWzaeqajmaOcqGHris5aaqcaaAaaOWaaabuaKaaGgaacqGF0oazkmaaBaaajeaObaGaem4zaCgabeaaaeaacqWGNbWzaeqajmaOcqGHris5aaaajaaOcqGGSaalaaa@6AC5@

with *δ*_*g *_an indicator for differentially expressed genes (see also Do *et al. *[13]). In the simulated data, we use *t*FDR and compare *t*FDR to *pe*FDR; for the experimental data, we have no choice but to use *pe*FDR.

### Simulation results for two studies

**Figure 1:**
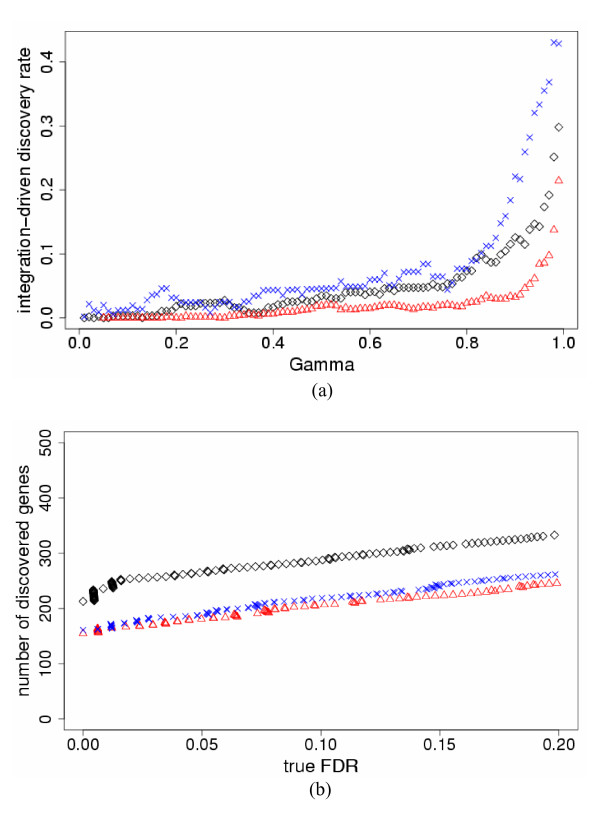
**IDR and discovered genes versus *t*FDR for the two-study simulation data**. a) Integration-driven discovery rate (IDR) versus threshold values of posterior probabilities of differential expression, *γ*, for the two-study simulated data and percent of differentially expressed genes *p *= 5% (blue checks), 10% (black diamonds), 25% (red triangles); b) The maximum number of differentially expressed genes versus true false discovery rate (*t*FDR) for individual analyses of Study 1 (red triangles), Study 2 (blue checks) and pooled analysis (black diamonds), for two-study simulated data and percent of differentially expressed genes *p *= 10%.

We simulated data for two studies, Study 1 and Study 2, with similar format to the *B. subtilis *mutant and induction studies, using three different values for the percent of truly differentially expressed genes: *p *= 5%, 10% and 25% (see Methods). We implemented Model (1) for each of the two simulation studies separately and in a pooled analysis. The IDR ranged from 1.9% to 42.9% for all values of *p *for *γ *≥ 50%, with maximum *t*FDR of 5% for the pooled analysis (Table 1). Note that *t*FDR is low for large values of *γ *due to the simulation procedure. The IDR increases as *γ *increases. IDR is also smaller for larger values of *p *(Figure 1a); this is due to the larger variability between studies. As a result, fewer genes have *D*_*g *_less than *γ *in both studies separately, which reduces IDR. In addition to identifying highly expressed genes by choosing a *γ *threshold, researchers often choose a maximum *t*FDR and examine lists of differentially expressed genes with corresponding *t*FDR. In Figure 1b, we display all *t*FDR levels < 20% for *p *= 10% and show the number of discovered genes for the two individual studies and the pooled analysis. This plot shows the considerable increase in the number of differentially expressed genes found in the pooled analysis versus the separate analyses for the same level of *t*FDR.

In addition to choosing a threshold value of *D*_*g *_or FDR, researchers are often interested in the top set of genes only, i.e. the top 300 genes. For this reason, we rank the genes based on *D*_*g *_in both the pooled and individual analyses and compare the resulting numbers of differentially expressed genes that are included in the top genes. For each of the three simulation studies, *p *= 5%, 10%, 25%, we choose a threshold of the top *p*% of genes. We find that the pooled model always identifies a larger number of differentially expressed genes than individual studies (Table 2).

**Figure 2:**
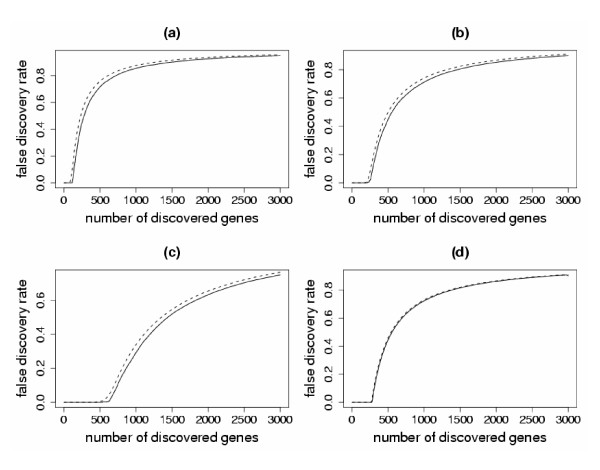
**True false discovery rate versus posterior expected false discovery rate for the simulation data**. True false discovery rate (*t*FDR) (solid lines) and posterior expected false discovery rate (*pe*FDR) (dashed lines) versus the number of discovered genes for: a) two-study simulation data, *p *= 5%; b) two-study simulation data, *p *= 10%; c) two-study simulation data, *p *= 25%; d) five-study simulation data, *p *= 10%.

We also compared *pe*FDR to *t*FDR for the simulation data to ensure that our *pe*FDR is a reasonable approximation to the true values. As seen from Figure 2, which displays all values of *pe*FDR versus *t*FDR for the simulation results, the *pe*FDR was always larger than *t*FDR, so that *pe*FDR is a conservative estimate of *t*FDR. The average differences between *pe*FDR and *t*FDR were less than 2.3% for all pooled simulation results. The maximum difference between *pe*FDR and *t*FDR decreased as the simulated percent of truly differentially expressed genes *p *increased. Specifically, for *p *= 5%, the average difference between *pe*FDR and *t*FDR was 2.3%, with maximum difference of 12.6% at *t*FDR = 19.9%. For *p *= 10%, the average difference was 2.2%, with maximum difference of 7.8% at *t*FDR = 3.1%. For *p *= 25%, the average difference was 2.3%, with maximum difference of 5.1% at *t*FDR = 23.8%.

### Simulation results for five studies

**Figure 3:**
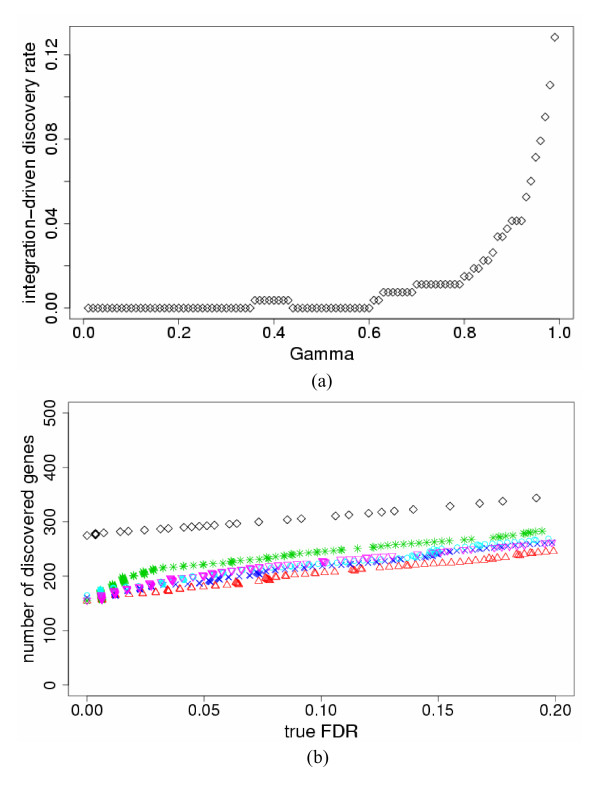
**IDR and discovered genes versus *t*FDR for the five-study simulation data**. a) Integration-driven discovery rate (IDR) versus threshold values of posterior probabilities of differential expression, *γ*, for the five-study simulated data and percent of differentially expressed genes *p *= 10%; b) The maximum number of differentially expressed genes versus true false discovery rate (*t*FDR) for individual analyses of Study 1 (red triangles), Study 2 (blue checks), Study 3 (green stars), Study 4 (turquoise circles), Study 5 (pink inverted triangles) and pooled analysis (black diamonds), for five-study simulated data and percent of differentially expressed genes *p *= 10%.

We also assessed our model for a meta-analysis with five studies. For this, we used the same Study 1 and Study 2 as in the previous section, and *p *= 10%. We then simulated three further studies similar to Study 1, but with different model parameters for ηjg02
 MathType@MTEF@5@5@+=feaafiart1ev1aaatCvAUfKttLearuWrP9MDH5MBPbIqV92AaeXatLxBI9gBaebbnrfifHhDYfgasaacH8akY=wiFfYdH8Gipec8Eeeu0xXdbba9frFj0=OqFfea0dXdd9vqai=hGuQ8kuc9pgc9s8qqaq=dirpe0xb9q8qiLsFr0=vr0=vr0dc8meaabaqaciaacaGaaeqabaqabeGadaaakeaaiiGacqWF3oaAdaqhaaWcbaGaemOAaOMaem4zaCMaeGimaadabaGaeGOmaidaaaaa@3320@,*c*, and variation over slides and experiments (see Methods). The IDR was 7.1% for *γ *= 0.95, and 12.8% for *γ *= 99%, with *t*FDR of 0% in the pooled analysis for these levels of *γ*. The IDR was lower for the same levels of *γ *for the five-study versus two-study pooled analysis. This was due to the larger variation between the five studies, resulting in fewer genes with *D*_*g *_less than *γ *in all studies, which reduced IDR. We plot IDR versus *γ *in Figure 3a. Figure 3b displays the number of discovered genes for the pooled analysis versus the five separate analyses for *t*FDR < 20%, which again shows a considerable increase for the pooled analysis.

We also show the number of differentially expressed genes identified by the pooled versus individual analyses for a fixed value of top expressed genes in Table 2. For the top 300 genes, the pooled model again identifies more differentially expressed genes than individual studies. We also compared *pe*FDR to *t*FDR in Figure 2d. The average difference was 0.54%, with maximum difference of 2.7%at *t*FDR = 0.36%. These values are smaller than the results for the two-study simulations, showing improved accuracy of *pe*FDR when pooling more data.

### Experimental data results

**Figure 4:**
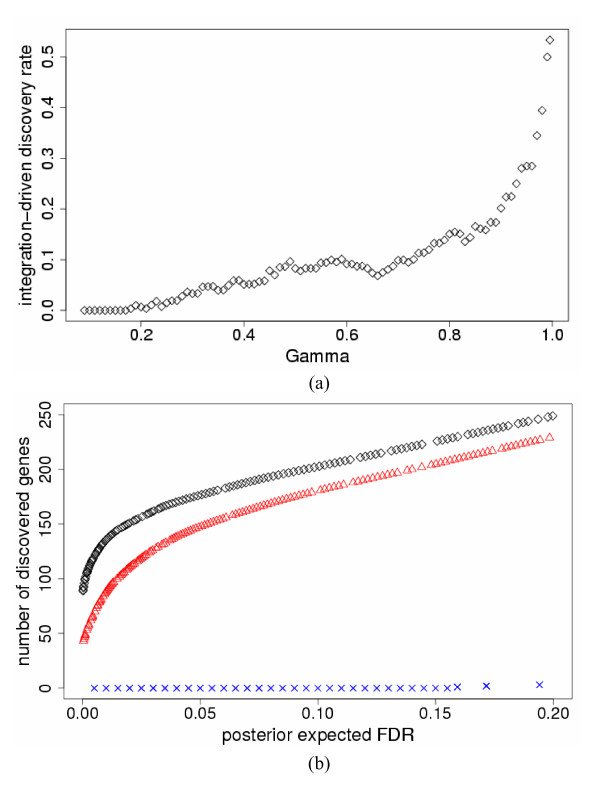
**IDR and discovered genes versus *pe*FDR for the experimental data**. a) Integration-driven discovery rate (IDR) versus threshold values of posterior probabilities of differential expression, *γ*, for the *B. subtilis *mutant and induction experimental study data; b) The maximum number of differentially expressed genes versus posterior expected false discovery rate (*pe*FDR) for individual analyses of the *B. subtilis *mutant study (red triangles), induction study (blue checks) and pooled analysis (black diamonds).

We implemented Model (1) to pool the mutant and induction *B. subtilis *studies, with 2,515 genes that had expression in both studies. We also implemented Model (1) for each study individually. For values of *γ *≥ 50% and maximum *pe*FDR of 11.5% for the pooled analysis, the IDR ranged from 8.2% to 53.3% (Table 3). We plot IDR versus *γ *in Figure 4a. Figure 4b presents the number of discovered genes for *pe*FDR < 20% for both the separate and pooled analyses. The induction study had much lower log-ratios of expression than the mutant study; the average 97.5%-ile for the induction experiments was 0.65 versus 1.59 for the mutant experiments. As a result, the maximum *D*_*g *_value for the induction study was 0.84, with minimum corresponding *pe*FDR of 16%. In contrast, the mutant study had 33 genes with *D*_*g *_of 1.0. Even though the values of *D*_*g *_were lower for the induction than the mutant study, we found that combining the two data sets resulted in more discoveries of differentially expressed genes than either study alone for fixed levels of *pe*FDR.

## Conclusion

We demonstrated here the usefulness of a Bayesian hierarchical model for pooling data across independent microarray studies with several sources of variation. The pooled method provides a systematic analysis framework, producing probability estimates of differential expression for each gene. These estimates are used to rank genes, calculate IDR, and produce posterior expected FDR values.

In the simulation of two and five studies, we found an appreciable increase in the IDR for various thresholds of the probability of differential expression, with corresponding low levels of *t*FDR. When fixing *t*FDR, we found more genes discovered in the pooled analysis than the separate analyses. When setting a threshold for the top genes of interest, the pooled model identified more truly differentially expressed genes than individual analyses. In the simulation of five studies, the IDR was somewhat lower than for two studies, but was still considerable. When comparing the *pe*FDR to *t*FDR in simulations, we found reasonable agreement, with *pe*FDR overestimating *t*FDR on average by less than 3%. The difference between *pe*FDR and *t*FDR decreased for the simulation of five studies, indicating that pooling more data improves the posterior estimation of FDR. In our analysis of experimental data, the IDR was also large. One study had somewhat lower probabilities of differential expression, which resulted in more discoveries when the data was pooled. We conclude that combining information across studies strengthens the probabilities of differential expression, improves IDR, and increases the number of discovered genes for fixed *t*FDR, *pe*FDR and fixed top percent of genes than individual study analyses.

Our model is designed for studies from the same platform. In the *B. subtilis *experimental data, a common control sample was used for the mutant and induction studies. However, our model does not require a common reference sample across studies, and assumes the studies are independent. In addition, all studies do not need to have the same array-design layout. This is due to the studies being linked only through the common parameter of differential expression, *p*; no other parameters are shared between studies. For example, one study could have only replicate slides, and another study could have both replicate slides and replicate experiments. We also assume that there are either no outlying studies or that the data has been scaled across studies before analysis. Future work will address the issues of pooling studies from different platforms and sets of studies that may contain outliers.

## Methods

### Simulation data for two studies

We simulated data for two studies, with the same format as the *B. subtilis *mutant and induction experimental data (see Methods: Experimental data), with the percent of differentially expressed genes of *p *= 5%, 10% and 25%. Each study had 3,000 genes; the first study had 5 replicate slides within 3 replicate experiments, and the second study had 4 replicate slides within 3 replicate experiments. We simulated data from Model (1), with parameters similar to those found in the experimental data. For Study 1, we used ηjg02
 MathType@MTEF@5@5@+=feaafiart1ev1aaatCvAUfKttLearuWrP9MDH5MBPbIqV92AaeXatLxBI9gBaebbnrfifHhDYfgasaacH8akY=wiFfYdH8Gipec8Eeeu0xXdbba9frFj0=OqFfea0dXdd9vqai=hGuQ8kuc9pgc9s8qqaq=dirpe0xb9q8qiLsFr0=vr0=vr0dc8meaabaqaciaacaGaaeqabaqabeGadaaakeaaiiGacqWF3oaAdaqhaaWcbaGaemOAaOMaem4zaCMaeGimaadabaGaeGOmaidaaaaa@3320@ = 0.015, and *c *= 66.67. The variance across slides was set to 0.074, and across experiments to 0.029. For Study 2, we used ηjg02
 MathType@MTEF@5@5@+=feaafiart1ev1aaatCvAUfKttLearuWrP9MDH5MBPbIqV92AaeXatLxBI9gBaebbnrfifHhDYfgasaacH8akY=wiFfYdH8Gipec8Eeeu0xXdbba9frFj0=OqFfea0dXdd9vqai=hGuQ8kuc9pgc9s8qqaq=dirpe0xb9q8qiLsFr0=vr0=vr0dc8meaabaqaciaacaGaaeqabaqabeGadaaakeaaiiGacqWF3oaAdaqhaaWcbaGaemOAaOMaem4zaCMaeGimaadabaGaeGOmaidaaaaa@3320@ = 0.02, and *c *= 40. The variance across slides was set to 0.02, and across experiments to 0.026.

Between study variance for the experimental data was 0.067 for all 2,515 genes, and 0.296 for the top 10% of genes. The simulated data had similar between study variance for all genes, and higher variability for the top genes than the experimental data. The between study variance was 0.053 for *p *= 5%, 0.105 for *p *= 10% and 0.23 for *p *= 25% for all 3,000 genes, with between study variance for the top genes of 0.714 for *p *= 5%, 0.887 for *p *= 10% and 0.86 for *p *= 25%. For each gene, log-expression ratios are simulated from normal distributions, independently of other genes. Although expression is expected to have some correlation among genes, this is difficult to model, and we thus assume independence for simulation purposes. The independence assumption was also used in simulation studies by other authors (see, for example, [8,9]).

### Simulation data for five studies

For the simulation of five studies, we used the Study 1 and Study 2 data from the previous section, and simulated data for 3 additional studies, with *p *= 10% for all studies. For Studies 3, 4 and 5, we simulated 5 replicate slides within 3 replicate experiments. For the parameters ηjg02
 MathType@MTEF@5@5@+=feaafiart1ev1aaatCvAUfKttLearuWrP9MDH5MBPbIqV92AaeXatLxBI9gBaebbnrfifHhDYfgasaacH8akY=wiFfYdH8Gipec8Eeeu0xXdbba9frFj0=OqFfea0dXdd9vqai=hGuQ8kuc9pgc9s8qqaq=dirpe0xb9q8qiLsFr0=vr0=vr0dc8meaabaqaciaacaGaaeqabaqabeGadaaakeaaiiGacqWF3oaAdaqhaaWcbaGaemOAaOMaem4zaCMaeGimaadabaGaeGOmaidaaaaa@3320@* ,c *and variance across slides and experiments, we used a range of values that were either between the values for Study 1 and Study 2, or somewhat larger or smaller than these two studies. For the Study 3, we used ηjg02
 MathType@MTEF@5@5@+=feaafiart1ev1aaatCvAUfKttLearuWrP9MDH5MBPbIqV92AaeXatLxBI9gBaebbnrfifHhDYfgasaacH8akY=wiFfYdH8Gipec8Eeeu0xXdbba9frFj0=OqFfea0dXdd9vqai=hGuQ8kuc9pgc9s8qqaq=dirpe0xb9q8qiLsFr0=vr0=vr0dc8meaabaqaciaacaGaaeqabaqabeGadaaakeaaiiGacqWF3oaAdaqhaaWcbaGaemOAaOMaem4zaCMaeGimaadabaGaeGOmaidaaaaa@3320@ = 0.017, and *c *= 70.6. The variance across slides was set to 0.05, and across experiments to 0.02. For Study 4, we used ηjg02
 MathType@MTEF@5@5@+=feaafiart1ev1aaatCvAUfKttLearuWrP9MDH5MBPbIqV92AaeXatLxBI9gBaebbnrfifHhDYfgasaacH8akY=wiFfYdH8Gipec8Eeeu0xXdbba9frFj0=OqFfea0dXdd9vqai=hGuQ8kuc9pgc9s8qqaq=dirpe0xb9q8qiLsFr0=vr0=vr0dc8meaabaqaciaacaGaaeqabaqabeGadaaakeaaiiGacqWF3oaAdaqhaaWcbaGaemOAaOMaem4zaCMaeGimaadabaGaeGOmaidaaaaa@3320@ = 0.02, and *c *= 55. The variance across slides was set to 0.04, and across experiments to 0.022. For Study 5, we used ηjg02
 MathType@MTEF@5@5@+=feaafiart1ev1aaatCvAUfKttLearuWrP9MDH5MBPbIqV92AaeXatLxBI9gBaebbnrfifHhDYfgasaacH8akY=wiFfYdH8Gipec8Eeeu0xXdbba9frFj0=OqFfea0dXdd9vqai=hGuQ8kuc9pgc9s8qqaq=dirpe0xb9q8qiLsFr0=vr0=vr0dc8meaabaqaciaacaGaaeqabaqabeGadaaakeaaiiGacqWF3oaAdaqhaaWcbaGaemOAaOMaem4zaCMaeGimaadabaGaeGOmaidaaaaa@3320@ = 0.015, and *c *= 60. The variance across slides was set to 0.06, and across experiments to 0.03. Between study variance ranged from 0.098 to 0.153 for all 5 studies for all 3,000 genes, and from 0.827 to 1.35 for the top 10% of genes, which was higher than the two-study experimental data.

### Experimental data

The *B. subtilis *experiments were designed to identify sporulation genes under the control of sigma factor E (*σ*^E^). Two complementary experimental setups were used, the first was a deletion of *σ*^E ^(mutant study) and the second an overexpression of *σ*^E ^(induction study), described in the following (for additional details, see [35,42]).

#### Mutant study

In the mutant study, the treated sample contained sporulating cells with a null mutation in the gene for *σ*^E ^(i.e. the mutant sample), and the control sample contained sporulating cells that were wild type for *σ*^E^. The wild-type/mutant ratios were examined; up-regulated genes were identified as belonging to the *σ*^E ^regulon. In total, five microarrays were produced from three independent identical experiments; the first experiment had three replicate arrays and the second and third experiments each had one array. The number of genes spotted on the five arrays ranged from 4,268 to 4,751; these values are larger than the *B. subtilis *genome size of 4,106 due to multiple spotting of selected genes on various arrays. The percent of low quality spots that were removed from analysis ranged from 18.6% to 64.5% of values across the five arrays. In total, there were 3,713 genes with measurable expression ratios in at least one microarray. Here, we analyze values after normalization using a rank-invariant method [2,43].

#### Induction study

In the induction study, *σ*^E ^was overexpressed in response to an inducer, i.e. cells that had been treated with an inducer were compared to control cells. The induction/wild-type ratios were examined; up-regulated genes were identified as belonging to the *σ*^E ^regulon. In total, four microarrays were produced from three independent identical experiments. The first two experiments each had one array, and the third experiment had two replicate arrays. The number of genes spotted on the four arrays ranged from 4,608 to 4,751; the percentage of genes detected on the arrays ranged from 33.0% to 47.4%. In total, there were 2,552 genes with measurable expression ratios in at least one microarray. Here, we again analyze the post-normalized values.

### Markov chain Monte Carlo implementation

In the Markov chain Monte Carlo analysis, the full conditionals are simulated as follows.

#### Joint posterior distribution

For the hierarchical model of (1), the joint distribution of the data and parameters is:

p(yjgse,μjge,τjg2,σjg2,θjg,Ig,p,ηjg02,cj)=∏j=1J∏g=1G∏e=1E{∏s=1Sep(yjgse|μjge,τjg2)p(μjge|θjg,σjg2)}p(θjg,Ig|p,Ωj)p(τjg2)p(σjg2)p(p)p(Ωj),
 MathType@MTEF@5@5@+=feaafiart1ev1aaatCvAUfKttLearuWrP9MDH5MBPbIqV92AaeXatLxBI9gBamXvP5wqSXMqHnxAJn0BKvguHDwzZbqegyvzYrwyUfgarqqtubsr4rNCHbGeaGqiA8vkIkVAFgIELiFeLkFeLk=iY=Hhbbf9v8qqaqFr0xc9pk0xbba9q8WqFfeaY=biLkVcLq=JHqVepeea0=as0db9vqpepesP0xe9Fve9Fve9GapdbaqaaeGacaGaaiaabeqaamqadiabaaGceaqabeaajaaOcqWGWbaCcqGGOaakcqWG5bqEkmaaBaaajeaObaGaemOAaOMaem4zaCMaem4CamNaemyzaugabeaajaaOcqGGSaaliiGacqWF8oqBkmaaBaaajeaObaGaemOAaOMaem4zaCMaemyzaugabeaajaaOcqGGSaalcqWFepaDkmaaDaaajeaObaGaemOAaOMaem4zaCgabaGaeGOmaidaaKaaGkabcYcaSiab=n8aZPWaa0baaKqaGgaacqWGQbGAcqWGNbWzaeaacqaIYaGmaaqcaaQaeiilaWIae8hUdeNcdaWgaaqcbaAaaiabdQgaQjabdEgaNbqabaqcaaQaeiilaWIaemysaKKcdaWgaaqcbaAaaiabdEgaNbqabaqcaaQaeiilaWIaemiCaaNaeiilaWIae83TdGMcdaqhaaqcbaAaaiabdQgaQjabdEgaNjabicdaWaqaaiabikdaYaaajaaOcqGGSaalcqWGJbWykmaaBaaajeaObaGaemOAaOgabeaajaaOcqGGPaqkcqGH9aqpaOqaamaarahajaaObaGcdaqeWbqcaaAaaOWaaebCaKaaGgaakmaacmaajaaObaGcdaqeWbqcaaAaaiabdchaWjabcIcaOiabdMha5PWaaSbaaKqaGgaacqWGQbGAcqWGNbWzcqWGZbWCcqWGLbqzaeqaaKaaGkabcYha8jab=X7aTPWaaSbaaKqaGgaacqWGQbGAcqWGNbWzcqWGLbqzaeqaaKaaGkabcYcaSiab=r8a0PWaa0baaKqaGgaacqWGQbGAcqWGNbWzaeaacqaIYaGmaaqcaaQaeiykaKIaemiCaaNaeiikaGIae8hVd0McdaWgaaqcbaAaaiabdQgaQjabdEgaNjabdwgaLbqabaqcaaQaeiiFaWNae8hUdeNcdaWgaaqcbaAaaiabdQgaQjabdEgaNbqabaqcaaQaeiilaWIae83WdmNcdaqhaaqcbaAaaiabdQgaQjabdEgaNbqaaiabikdaYaaajaaOcqGGPaqkaKqaGgaacqWGZbWCcqGH9aqpcqaIXaqmaeaacqWGtbWulmaaBaaajiaObaGaemyzaugabeaaaKWaGkabg+GivdaajaaOcaGL7bGaayzFaaGaemiCaaNaeiikaGIae8hUdeNcdaWgaaqcbaAaaiabdQgaQjabdEgaNbqabaqcaaQaeiilaWIaemysaKKcdaWgaaqcbaAaaiabdEgaNbqabaqcaaQaeiiFaWNaemiCaaNaeiilaWccceGae4xQdCLcdaWgaaqcbaAaaGWabiaa9PgaaeqaaKaaGkabcMcaPaqcbaAaaiabdwgaLjabg2da9iabigdaXaqaaiabdweafbqcdaQaey4dIunajaaOcqWGWbaCcqGGOaakcqWFepaDkmaaDaaajeaObaGaemOAaOMaem4zaCgabaGaeGOmaidaaKaaGkabcMcaPiabdchaWbqcbaAaaiabdEgaNjabg2da9iabigdaXaqaaiabdEeahbqcdaQaey4dIunajaaOcqGGOaakcqWFdpWCkmaaDaaajeaObaGaemOAaOMaem4zaCgabaGaeGOmaidaaKaaGkabcMcaPiabdchaWjabcIcaOiabdchaWjabcMcaPiabdchaWjabcIcaOiab+L6axPWaaSbaaKqaGgaacaqFQbaabeaajaaOcqGGPaqkaKqaGgaacqWGQbGAcqGH9aqpcqaIXaqmaeaacqWGkbGsaKWaGkabg+GivdqcaaQaeiilaWcaaaa@12D6@

where **Ω**_**j **_= (ηjg02
 MathType@MTEF@5@5@+=feaafiart1ev1aaatCvAUfKttLearuWrP9MDH5MBPbIqV92AaeXatLxBI9gBaebbnrfifHhDYfgasaacH8akY=wiFfYdH8Gipec8Eeeu0xXdbba9frFj0=OqFfea0dXdd9vqai=hGuQ8kuc9pgc9s8qqaq=dirpe0xb9q8qiLsFr0=vr0=vr0dc8meaabaqaciaacaGaaeqabaqabeGadaaakeaaiiGacqWF3oaAdaqhaaWcbaGaemOAaOMaem4zaCMaeGimaadabaGaeGOmaidaaaaa@3320@, *c*_*j*_), *j *= study, *g *= gene, *e *= experiment, *s *= slide.

#### Prior distributions

The prior distributions are specified as follow.

τjg2~kτ˜j2/χk2σjg2~hσ˜j2/χh2
 MathType@MTEF@5@5@+=feaafiart1ev1aaatCvAUfKttLearuWrP9MDH5MBPbIqV92AaeXatLxBI9gBaebbnrfifHhDYfgasaacH8akY=wiFfYdH8Gipec8Eeeu0xXdbba9frFj0=OqFfea0dXdd9vqai=hGuQ8kuc9pgc9s8qqaq=dirpe0xb9q8qiLsFr0=vr0=vr0dc8meaabaqaciaacaGaaeqabaqabeGadaaakqaabeqaaGGaciab=r8a0naaDaaaleaacqWGQbGAcqWGNbWzaeaacqaIYaGmaaGccqGG+bGFcqWGRbWAcuWFepaDgaacamaaDaaaleaacqWGQbGAaeaacqaIYaGmaaGccqGGVaWlcqWFhpWydaqhaaWcbaGaem4AaSgabaGaeGOmaidaaaGcbaGae83Wdm3aa0baaSqaaiabdQgaQjabdEgaNbqaaiabikdaYaaakiabc6ha+jabdIgaOjqb=n8aZzaaiaWaa0baaSqaaiabdQgaQbqaaiabikdaYaaakiabc+caViab=D8aJnaaDaaaleaacqWGObaAaeaacqaIYaGmaaaaaaa@508F@

Here, (χk2)−1
 MathType@MTEF@5@5@+=feaafiart1ev1aaatCvAUfKttLearuWrP9MDH5MBPbIqV92AaeXatLxBI9gBaebbnrfifHhDYfgasaacH8akY=wiFfYdH8Gipec8Eeeu0xXdbba9frFj0=OqFfea0dXdd9vqai=hGuQ8kuc9pgc9s8qqaq=dirpe0xb9q8qiLsFr0=vr0=vr0dc8meaabaqaciaacaGaaeqabaqabeGadaaakeaacqGGOaakiiGacqWFhpWydaqhaaWcbaGaem4AaSgabaGaeGOmaidaaOGaeiykaKYaaWbaaSqabeaacqGHsislcqaIXaqmaaaaaa@34AE@ denotes the standard inverse *χ*^2 ^distribution with *k *degrees of freedom, and τ˜j2
 MathType@MTEF@5@5@+=feaafiart1ev1aaatCvAUfKttLearuWrP9MDH5MBPbIqV92AaeXatLxBI9gBaebbnrfifHhDYfgasaacH8akY=wiFfYdH8Gipec8Eeeu0xXdbba9frFj0=OqFfea0dXdd9vqai=hGuQ8kuc9pgc9s8qqaq=dirpe0xb9q8qiLsFr0=vr0=vr0dc8meaabaqaciaacaGaaeqabaqabeGadaaakeaaiiGacuWFepaDgaacamaaDaaaleaacqWGQbGAaeaacqaIYaGmaaaaaa@3103@ and σ˜j2
 MathType@MTEF@5@5@+=feaafiart1ev1aaatCvAUfKttLearuWrP9MDH5MBPbIqV92AaeXatLxBI9gBaebbnrfifHhDYfgasaacH8akY=wiFfYdH8Gipec8Eeeu0xXdbba9frFj0=OqFfea0dXdd9vqai=hGuQ8kuc9pgc9s8qqaq=dirpe0xb9q8qiLsFr0=vr0=vr0dc8meaabaqaciaacaGaaeqabaqabeGadaaakeaaiiGacuWFdpWCgaacamaaDaaaleaacqWGQbGAaeaacqaIYaGmaaaaaa@3101@ are scale parameters of the inverse chi-squared distribution derived from the data. τ˜j2
 MathType@MTEF@5@5@+=feaafiart1ev1aaatCvAUfKttLearuWrP9MDH5MBPbIqV92AaeXatLxBI9gBaebbnrfifHhDYfgasaacH8akY=wiFfYdH8Gipec8Eeeu0xXdbba9frFj0=OqFfea0dXdd9vqai=hGuQ8kuc9pgc9s8qqaq=dirpe0xb9q8qiLsFr0=vr0=vr0dc8meaabaqaciaacaGaaeqabaqabeGadaaakeaaiiGacuWFepaDgaacamaaDaaaleaacqWGQbGAaeaacqaIYaGmaaaaaa@3103@ is produced as follows:

τ˜j2=1G(∑Se−1)∑g=1G∑e=1E∑s=1Se(yjgse−yjg⋅e)2,
 MathType@MTEF@5@5@+=feaafiart1ev1aaatCvAUfKttLearuWrP9MDH5MBPbIqV92AaeXatLxBI9gBaebbnrfifHhDYfgasaacH8akY=wiFfYdH8Gipec8Eeeu0xXdbba9frFj0=OqFfea0dXdd9vqai=hGuQ8kuc9pgc9s8qqaq=dirpe0xb9q8qiLsFr0=vr0=vr0dc8meaabaqaciaacaGaaeqabaqabeGadaaakeaaiiGacuWFepaDgaacamaaDaaaleaacqWGQbGAaeaacqaIYaGmaaGccqGH9aqpdaWcaaqaaiabigdaXaqaaiabdEeahnaabmaabaWaaabqaeaacqWGtbWudaWgaaWcbaGaemyzaugabeaakiabgkHiTiabigdaXaWcbeqab0GaeyyeIuoaaOGaayjkaiaawMcaaaaadaaeWbqaamaaqahabaWaaabCaeaadaqadaqaaiabdMha5naaBaaaleaacqWGQbGAcqWGNbWzcqWGZbWCcqWGLbqzaeqaaOGaeyOeI0IaemyEaK3aaSbaaSqaaiabdQgaQjabdEgaNjabgwSixlabdwgaLbqabaaakiaawIcacaGLPaaadaahaaWcbeqaaiabikdaYaaaaeaacqWGZbWCcqGH9aqpcqaIXaqmaeaacqWGtbWudaWgaaadbaGaemyzaugabeaaa0GaeyyeIuoaaSqaaiabdwgaLjabg2da9iabigdaXaqaaiabdweafbqdcqGHris5aaWcbaGaem4zaCMaeyypa0JaeGymaedabaGaem4raCeaniabggHiLdGccqGGSaalaaa@659D@

where *y*_*jg*.*e *_is the average log-ratio of expression over the slides within an experiment:

yjg⋅e=1Se∑s=1Seyjgse.
 MathType@MTEF@5@5@+=feaafiart1ev1aaatCvAUfKttLearuWrP9MDH5MBPbIqV92AaeXatLxBI9gBaebbnrfifHhDYfgasaacH8akY=wiFfYdH8Gipec8Eeeu0xXdbba9frFj0=OqFfea0dXdd9vqai=hGuQ8kuc9pgc9s8qqaq=dirpe0xb9q8qiLsFr0=vr0=vr0dc8meaabaqaciaacaGaaeqabaqabeGadaaakeaacqWG5bqEdaWgaaWcbaGaemOAaOMaem4zaCMaeyyXICTaemyzaugabeaakiabg2da9maalaaabaGaeGymaedabaGaem4uam1aaSbaaSqaaiabdwgaLbqabaaaaOWaaabCaeaacqWG5bqEdaWgaaWcbaGaemOAaOMaem4zaCMaem4CamNaemyzaugabeaaaeaacqWGZbWCcqGH9aqpcqaIXaqmaeaacqWGtbWudaWgaaadbaGaemyzaugabeaaa0GaeyyeIuoakiabc6caUaaa@49C2@

Similarly, the scale parameter for σjg2
 MathType@MTEF@5@5@+=feaafiart1ev1aaatCvAUfKttLearuWrP9MDH5MBPbIqV92AaeXatLxBI9gBaebbnrfifHhDYfgasaacH8akY=wiFfYdH8Gipec8Eeeu0xXdbba9frFj0=OqFfea0dXdd9vqai=hGuQ8kuc9pgc9s8qqaq=dirpe0xb9q8qiLsFr0=vr0=vr0dc8meaabaqaciaacaGaaeqabaqabeGadaaakeaaiiGacqWFdpWCdaqhaaWcbaGaemOAaOMaem4zaCgabaGaeGOmaidaaaaa@3249@ is calculated as follows:

σ˜j2=1G(E−1)∑g=1G∑e=1E(yjg.e−yjg..)2
 MathType@MTEF@5@5@+=feaafiart1ev1aaatCvAUfKttLearuWrP9MDH5MBPbIqV92AaeXatLxBI9gBaebbnrfifHhDYfgasaacH8akY=wiFfYdH8Gipec8Eeeu0xXdbba9frFj0=OqFfea0dXdd9vqai=hGuQ8kuc9pgc9s8qqaq=dirpe0xb9q8qiLsFr0=vr0=vr0dc8meaabaqaciaacaGaaeqabaqabeGadaaakeaaiiGacuWFdpWCgaacamaaDaaaleaacqWGQbGAaeaacqaIYaGmaaGccqGH9aqpdaWcaaqaaiabigdaXaqaaiabdEeahnaabmaabaGaemyrauKaeyOeI0IaeGymaedacaGLOaGaayzkaaaaamaaqahabaWaaabCaeaadaqadaqaaiabdMha5naaBaaaleaacqWGQbGAcqWGNbWzcqGGUaGlcqWGLbqzaeqaaOGaeyOeI0IaemyEaK3aaSbaaSqaaiabdQgaQjabdEgaNjabc6caUiabc6caUaqabaaakiaawIcacaGLPaaadaahaaWcbeqaaiabikdaYaaaaeaacqWGLbqzcqGH9aqpcqaIXaqmaeaacqWGfbqra0GaeyyeIuoaaSqaaiabdEgaNjabg2da9iabigdaXaqaaiabdEeahbqdcqGHris5aaaa@5638@

where y_*jg*.. _is the average log-ratio of expression over both slides and experiments. We use 3 degrees of freedom in each study for both τjg2
 MathType@MTEF@5@5@+=feaafiart1ev1aaatCvAUfKttLearuWrP9MDH5MBPbIqV92AaeXatLxBI9gBaebbnrfifHhDYfgasaacH8akY=wiFfYdH8Gipec8Eeeu0xXdbba9frFj0=OqFfea0dXdd9vqai=hGuQ8kuc9pgc9s8qqaq=dirpe0xb9q8qiLsFr0=vr0=vr0dc8meaabaqaciaacaGaaeqabaqabeGadaaakeaaiiGacqWFepaDdaqhaaWcbaGaemOAaOMaem4zaCgabaGaeGOmaidaaaaa@324B@ and σjg2
 MathType@MTEF@5@5@+=feaafiart1ev1aaatCvAUfKttLearuWrP9MDH5MBPbIqV92AaeXatLxBI9gBaebbnrfifHhDYfgasaacH8akY=wiFfYdH8Gipec8Eeeu0xXdbba9frFj0=OqFfea0dXdd9vqai=hGuQ8kuc9pgc9s8qqaq=dirpe0xb9q8qiLsFr0=vr0=vr0dc8meaabaqaciaacaGaaeqabaqabeGadaaakeaaiiGacqWFdpWCdaqhaaWcbaGaemOAaOMaem4zaCgabaGaeGOmaidaaaaa@3249@, i.e. *h *= *k *= 3. The prior distributions for the remaining parameters are as follow.

θjg|Ig=0~N(0,ηjg02)θjg|Ig=1~N(0,cj×ηjg02)Ig~Bernoulli(p)p~Uniform(0,1)ηjg02~as12/χa2cj~bs22/χb2
 MathType@MTEF@5@5@+=feaafiart1ev1aaatCvAUfKttLearuWrP9MDH5MBPbIqV92AaeXatLxBI9gBamXvP5wqSXMqHnxAJn0BKvguHDwzZbqegyvzYrwyUfgarqqtubsr4rNCHbGeaGqiA8vkIkVAFgIELiFeLkFeLk=iY=Hhbbf9v8qqaqFr0xc9pk0xbba9q8WqFfeaY=biLkVcLq=JHqVepeea0=as0db9vqpepesP0xe9Fve9Fve9GapdbaqaaeGacaGaaiaabeqaamqadiabaaGcbaqcaaAbaeaabyWaaaaabaacciGae8hUdeNcdaWgaaqcbaAaaiabdQgaQjabdEgaNbqabaqcaaQaeiiFaWNaemysaKKcdaWgaaqcbaAaaiabdEgaNbqabaqcaaQaeyypa0JaeGimaadabaGaeiOFa4habaGaeeOta4KaeeikaGIaeGimaaJaeiilaWIae83TdGMcdaqhaaqcbaAaaiabdQgaQjabdEgaNjabicdaWaqaaiabikdaYaaajaaOcqGGPaqkaeaacqWF4oqCkmaaBaaajeaObaGaemOAaOMaem4zaCgabeaajaaOcqGG8baFcqWGjbqskmaaBaaajeaObaGaem4zaCgabeaajaaOcqGH9aqpcqaIXaqmaeaacqGG+bGFaeaacqqGobGtcqqGOaakcqaIWaamcqGGSaalcqWGJbWykmaaBaaajeaObaGaemOAaOgabeaajaaOcqGHxdaTcqWF3oaAkmaaDaaajeaObaGaemOAaOMaem4zaCMaeGimaadabaGaeGOmaidaaKaaGkabcMcaPaqaceaaizGaaCzcaiabdMeajPWaaSbaaKqaGgaacqWGNbWzaeqaaaqcaaAaaiabc6ha+bqaaiabbkeacjabbwgaLjabbkhaYjabb6gaUjabb+gaVjabbwha1jabbYgaSjabbYgaSjabbMgaPPWaaeWaaKaaGgaacqWGWbaCaiaawIcacaGLPaaaaeGabaaWgiaaxMaacqWGWbaCaeaacqGG+bGFaeaacqqGvbqvcqqGUbGBcqqGPbqAcqqGMbGzcqqGVbWBcqqGYbGCcqqGTbqBkmaabmaajaaObaGaeGimaaJaeiilaWIaeGymaedacaGLOaGaayzkaaaabiqaaqtbcaWLjaGae83TdGMcdaqhaaqcbaAaaiabdQgaQjabdEgaNjabicdaWaqaaiabikdaYaaaaKaaGgaacqGG+bGFaeaacqWGHbqycqWGZbWCkmaaDaaajeaObaGaeGymaedabaGaeGOmaidaaKaaGkabc+caViab=D8aJPWaa0baaKqaGgaacqWGHbqyaeaacqaIYaGmaaaajaaObiqaaGKbcaWLjaGaem4yamMcdaWgaaqcbaAaaiabdQgaQbqabaaajaaObaGaeiOFa4habaGaemOyaiMaem4CamNcdaqhaaqcbaAaaiabikdaYaqaaiabikdaYaaajaaOcqGGVaWlcqWFhpWykmaaDaaajeaObaGaemOyaigabaGaeGOmaidaaaaaaaa@CC31@

We choose *a*, s12
 MathType@MTEF@5@5@+=feaafiart1ev1aaatCvAUfKttLearuWrP9MDH5MBPbIqV92AaeXatLxBI9gBaebbnrfifHhDYfgasaacH8akY=wiFfYdH8Gipec8Eeeu0xXdbba9frFj0=OqFfea0dXdd9vqai=hGuQ8kuc9pgc9s8qqaq=dirpe0xb9q8qiLsFr0=vr0=vr0dc8meaabaqaciaacaGaaeqabaqabeGadaaakeaacqWGZbWCdaqhaaWcbaGaeGymaedabaGaeGOmaidaaaaa@302A@ so that the prior mean of ηjg02
 MathType@MTEF@5@5@+=feaafiart1ev1aaatCvAUfKttLearuWrP9MDH5MBPbIqV92AaeXatLxBI9gBamXvP5wqSXMqHnxAJn0BKvguHDwzZbqegyvzYrwyUfgarqqtubsr4rNCHbGeaGqiA8vkIkVAFgIELiFeLkFeLk=iY=Hhbbf9v8qqaqFr0xc9pk0xbba9q8WqFfeaY=biLkVcLq=JHqVepeea0=as0db9vqpepesP0xe9Fve9Fve9GapdbaqaaeGacaGaaiaabeqaamqadiabaaGcbaacciqcaaQae83TdGMcdaqhaaqcbaAaaiabdQgaQjabdEgaNjabicdaWaqaaiabikdaYaaaaaa@44B1@ is 1 with variance 0.1. We choose *b*, s22
 MathType@MTEF@5@5@+=feaafiart1ev1aaatCvAUfKttLearuWrP9MDH5MBPbIqV92AaeXatLxBI9gBaebbnrfifHhDYfgasaacH8akY=wiFfYdH8Gipec8Eeeu0xXdbba9frFj0=OqFfea0dXdd9vqai=hGuQ8kuc9pgc9s8qqaq=dirpe0xb9q8qiLsFr0=vr0=vr0dc8meaabaqaciaacaGaaeqabaqabeGadaaakeaacqWGZbWCdaqhaaWcbaGaeGOmaidabaGaeGOmaidaaaaa@302C@ so that the prior mean of *c*_*j *_is 100 with variance 10,000.

#### Full conditional posterior distributions

Each parameter is sampled from the full conditional posterior distributions by the following.

μjge|rest~N(Seyjg⋅eσjg2+τjg2θjgSeσjg2+τjg2,τjg2σjg2Seσjg2+τjg2),τjg2|rest~{∑e=1E∑s=1Se(yjgse−μjge)2+kτ˜j2}/χS1+…+SE+k,2σjg2|rest~[∑e=1E(μjge−μjg⋅)2+hσ˜j2]/χE+h−12.
 MathType@MTEF@5@5@+=feaafiart1ev1aaatCvAUfKttLearuWrP9MDH5MBPbIqV92AaeXatLxBI9gBamXvP5wqSXMqHnxAJn0BKvguHDwzZbqegyvzYrwyUfgarqqtubsr4rNCHbGeaGqiA8vkIkVAFgIELiFeLkFeLk=iY=Hhbbf9v8qqaqFr0xc9pk0xbba9q8WqFfeaY=biLkVcLq=JHqVepeea0=as0db9vqpepesP0xe9Fve9Fve9GapdbaqaaeGacaGaaiaabeqaamqadiabaaGcbaqcaaAbaeaabmWaaaqaaGGaciab=X7aTPWaaSbaaKqaGgaacqWGQbGAcqWGNbWzcqWGLbqzaeqaaKaaGkabcYha8jabbkhaYjabbwgaLjabbohaZjabbsha0bqaaiabb6ha+bqaaiabd6eaoPWaaeWaaKaaGgaakmaalaaajaaObaGaem4uamLcdaWgaaqcbaAaaiabdwgaLbqabaqcaaQaemyEaKNcdaWgaaqcbaAaaiabdQgaQjabdEgaNjabgwSixlabdwgaLbqabaqcaaQae83WdmNcdaqhaaqcbaAaaiabdQgaQjabdEgaNbqaaiabikdaYaaajaaOcqGHRaWkcqWFepaDkmaaDaaajeaObaGaemOAaOMaem4zaCgabaGaeGOmaidaaKaaGkab=H7aXPWaaSbaaKqaGgaacqWGQbGAcqWGNbWzaeqaaaqcaaAaaiabdofatPWaaSbaaKqaGgaacqWGLbqzaeqaaKaaGkab=n8aZPWaa0baaKqaGgaacqWGQbGAcqWGNbWzaeaacqaIYaGmaaqcaaQaey4kaSIae8hXdqNcdaqhaaqcbaAaaiabdQgaQjabdEgaNbqaaiabikdaYaaaaaqcaaQaeiilaWIcdaWcaaqcaaAaaiab=r8a0PWaa0baaKqaGgaacqWGQbGAcqWGNbWzaeaacqaIYaGmaaqcaaQae83WdmNcdaqhaaqcbaAaaiabdQgaQjabdEgaNbqaaiabikdaYaaaaKaaGgaacqWGtbWukmaaBaaajeaObaGaemyzaugabeaajaaOcqWFdpWCkmaaDaaajeaObaGaemOAaOMaem4zaCgabaGaeGOmaidaaKaaGkabgUcaRiab=r8a0PWaa0baaKqaGgaacqWGQbGAcqWGNbWzaeaacqaIYaGmaaaaaaqcaaQaayjkaiaawMcaaiabcYcaSaqaciaa0caaueGaaCzcaiab=r8a0PWaa0baaKqaGgaacqWGQbGAcqWGNbWzaeaacqaIYaGmaaqcaaQaeiiFaWNaeeOCaiNaeeyzauMaee4CamNaeeiDaqhabaGaeeOFa4habaGcdaWcgaqcaaAaaOWaaiWaaKaaGgaakmaaqahajaaObaGcdaaeWbqcaaAaaiabbIcaOiabdMha5PWaaSbaaKqaGgaacqWGQbGAcqWGNbWzcqWGZbWCcqWGLbqzaeqaaKaaGkabgkHiTiab=X7aTPWaaSbaaKqaGgaacqWGQbGAcqWGNbWzcqWGLbqzaeqaaKaaGkabcMcaPOWaaWbaaKqaGgqabaGaeGOmaidaaKaaGkabgUcaRiabdUgaRjqb=r8a0zaaiaGcdaqhaaqcbaAaaiabdQgaQbqaaiabikdaYaaaaeaacqWGZbWCcqGH9aqpcqaIXaqmaeaacqWGtbWulmaaBaaajiaObaGaemyzaugabeaaaKWaGkabggHiLdaajeaObaGaemyzauMaeyypa0JaeGymaedabaGaemyraueajmaOcqGHris5aaqcaaQaay5Eaiaaw2haaaqaaiab=D8aJPWaa0baaKqaGgaacqWGtbWulmaaBaaajiaObaGaeGymaedabeaajeaOcqGHRaWkcqWIMaYscqGHRaWkcqWGtbWulmaaBaaajiaObaGaemyraueabeaajeaOcqGHRaWkcqWGRbWAcqGGSaalaeaacqaIYaGmaaaaaaqcaaAaceaaOcGaaCzcaiab=n8aZPWaa0baaKqaGgaacqWGQbGAcqWGNbWzaeaacqaIYaGmaaqcaaQaeiiFaWNaeeOCaiNaeeyzauMaee4CamNaeeiDaqhabaGaeeOFa4habaGcdaWcgaqcaaAaaOWaamWaaKaaGgaakmaaqahajaaObaGaeeikaGIae8hVd0McdaWgaaqcbaAaaiabdQgaQjabdEgaNjabdwgaLbqabaqcaaQaeyOeI0Iae8hVd0McdaWgaaqcbaAaaiabdQgaQjabdEgaNjabgwSixdqabaqcaaQaeiykaKIcdaahaaqcbaAabeaacqaIYaGmaaaabaGaemyzauMaeyypa0JaeGymaedabaGaemyraueajmaOcqGHris5aKaaGkabgUcaRiabdIgaOjqb=n8aZzaaiaGcdaqhaaqcbaAaaiabdQgaQbqaaiabikdaYaaaaKaaGkaawUfacaGLDbaaaeaacqWFhpWykmaaDaaajeaObaGaemyrauKaey4kaSIaemiAaGMaeyOeI0IaeGymaedabaGaeGOmaidaaaaajaaOcqGGUaGlaaaaaa@414A@

Here, χS1+…+SE+k2
 MathType@MTEF@5@5@+=feaafiart1ev1aaatCvAUfKttLearuWrP9MDH5MBPbIqV92AaeXatLxBI9gBaebbnrfifHhDYfgasaacH8akY=wiFfYdH8Gipec8Eeeu0xXdbba9frFj0=OqFfea0dXdd9vqai=hGuQ8kuc9pgc9s8qqaq=dirpe0xb9q8qiLsFr0=vr0=vr0dc8meaabaqaciaacaGaaeqabaqabeGadaaakeaaiiGacqWFhpWydaqhaaWcbaGaem4uam1aaSbaaWqaaiabigdaXaqabaWccqGHRaWkcqWIMaYscqGHRaWkcqWGtbWudaWgaaadbaGaemyraueabeaaliabgUcaRiabdUgaRbqaaiabikdaYaaaaaa@3981@ denotes the standard inverse *χ*^2 ^distribution with (*S*_*1 *_+...+ *S*_*E *_+ *k*) degrees of freedom, with scale parameter:

{∑e=1E∑s=1Se(yjgse−μjge)2+kτ˜j2}/(S1+…+SE+k).
 MathType@MTEF@5@5@+=feaafiart1ev1aaatCvAUfKttLearuWrP9MDH5MBPbIqV92AaeXatLxBI9gBamXvP5wqSXMqHnxAJn0BKvguHDwzZbqegyvzYrwyUfgarqqtubsr4rNCHbGeaGqiA8vkIkVAFgIELiFeLkFeLk=iY=Hhbbf9v8qqaqFr0xc9pk0xbba9q8WqFfeaY=biLkVcLq=JHqVepeea0=as0db9vqpepesP0xe9Fve9Fve9GapdbaqaaeGacaGaaiaabeqaamqadiabaaGcbaWaaSGbaKaaGgaakmaacmaajaaObaGcdaaeWbqcaaAaaOWaaabCaKaaGgaacqGGOaakcqWG5bqEkmaaBaaajeaObaGaemOAaOMaem4zaCMaem4CamNaemyzaugabeaajaaOcqGHsisliiGacqWF8oqBkmaaBaaajeaObaGaemOAaOMaem4zaCMaemyzaugabeaajaaOcqGGPaqkkmaaCaaajeaObeqaaiabikdaYaaajaaOcqGHRaWkcqWGRbWAcuWFepaDgaacaOWaa0baaKqaGgaacqWGQbGAaeaacqaIYaGmaaaabaGaem4CamNaeyypa0JaeGymaedabaGaem4uam1cdaWgaaqccaAaaiabdwgaLbqabaaajmaOcqGHris5aaqcbaAaaiabdwgaLjabg2da9iabigdaXaqaaiabdweafbqcdaQaeyyeIuoaaKaaGkaawUhacaGL9baaaeaacqGGOaakcqWGtbWukmaaBaaajeaObaGaeGymaedabeaajaaOcqGHRaWkcqWIMaYscqGHRaWkcqWGtbWukmaaBaaajeaObaGaemyraueabeaajaaOcqGHRaWkcqWGRbWAcqGGPaqkcqGGUaGlaaaaaa@7F1D@

For *I*_*g *_= 0, the following full conditionals are sampled:

θjg|Ig=0, rest ~N(Eηjg02μjg⋅Eηjg02+σjg2,σg2ηjg02Eηjg02+σjg2),ηjg02|Ig=0, rest~[as12+θjg2a+1]/χa+12.
 MathType@MTEF@5@5@+=feaafiart1ev1aaatCvAUfKttLearuWrP9MDH5MBPbIqV92AaeXatLxBI9gBamXvP5wqSXMqHnxAJn0BKvguHDwzZbqegyvzYrwyUfgarqqtubsr4rNCHbGeaGqiA8vkIkVAFgIELiFeLkFeLk=iY=Hhbbf9v8qqaqFr0xc9pk0xbba9q8WqFfeaY=biLkVcLq=JHqVepeea0=as0db9vqpepesP0xe9Fve9Fve9GapdbaqaaeGacaGaaiaabeqaamqadiabaaGceaqabeaaiiGajaaOcqWF4oqCkmaaBaaajeaObaGaemOAaOMaem4zaCgabeaajaaOcqGG8baFcqWGjbqskmaaBaaajeaObaGaem4zaCgabeaajaaOcqGH9aqpcqaIWaamcqGGSaalcqqGGaaicqqGYbGCcqqGLbqzcqqGZbWCcqqG0baDcqqGGaaicqGG+bGFcqWGobGtkmaabmaajaaObaGcdaWcaaqcaaAaaiabdweafjab=D7aOPWaa0baaKqaGgaacqWGQbGAcqWGNbWzcqaIWaamaeaacqaIYaGmaaqcaaQae8hVd0McdaWgaaqcbaAaaiabdQgaQjabdEgaNjabgwSixdqabaaajaaObaGaemyrauKae83TdGMcdaqhaaqcbaAaaiabdQgaQjabdEgaNjabicdaWaqaaiabikdaYaaajaaOcqGHRaWkcqWFdpWCkmaaDaaajeaObaGaemOAaOMaem4zaCgabaGaeGOmaidaaaaajaaOcqGGSaalkmaalaaajaaObaGae83WdmNcdaqhaaqcbaAaaiabdEgaNbqaaiabikdaYaaajaaOcqWF3oaAkmaaDaaajeaObaGaemOAaOMaem4zaCMaeGimaadabaGaeGOmaidaaaqcaaAaaiabdweafjab=D7aOPWaa0baaKqaGgaacqWGQbGAcqWGNbWzcqaIWaamaeaacqaIYaGmaaqcaaQaey4kaSIae83WdmNcdaqhaaqcbaAaaiabdQgaQjabdEgaNbqaaiabikdaYaaaaaaajaaOcaGLOaGaayzkaaGaeiilaWcakeaajaaOcqWF3oaAkmaaDaaajeaObaGaemOAaOMaem4zaCMaeGimaadabaGaeGOmaidaaKaaGkabcYha8jabdMeajPWaaSbaaKqaGgaacqWGNbWzaeqaaKaaGkabg2da9iabicdaWiabcYcaSiabbccaGiabbkhaYjabbwgaLjabbohaZjabbsha0jabc6ha+PWaaSGbaKaaGgaakmaadmaajaaObaGcdaWcaaqcaaAaaiabdggaHjabdohaZPWaa0baaKqaGgaacqaIXaqmaeaacqaIYaGmaaqcaaQaey4kaSIae8hUdeNcdaqhaaqcbaAaaiabdQgaQjabdEgaNbqaaiabikdaYaaaaKaaGgaacqWGHbqycqGHRaWkcqaIXaqmaaaacaGLBbGaayzxaaaabaGae83XdmMcdaqhaaqcbaAaaiabdggaHjabgUcaRiabigdaXaqaaiabikdaYaaaaaqcaaQaeiOla4caaaa@D056@

For *I*_*g *_= 1, the following full conditionals are sampled:

θjg|Ig=1, rest ~N(Ecjηjg02μjg⋅Ecjηjg02+σjg2,σjg2cjηjg02Ecjηjg02+σjg2),ηjg02|Ig=1, rest~[cjas12+θjg2cj(a+1)]/χa+12.
 MathType@MTEF@5@5@+=feaafiart1ev1aaatCvAUfKttLearuWrP9MDH5MBPbIqV92AaeXatLxBI9gBamXvP5wqSXMqHnxAJn0BKvguHDwzZbqegyvzYrwyUfgarqqtubsr4rNCHbGeaGqiA8vkIkVAFgIELiFeLkFeLk=iY=Hhbbf9v8qqaqFr0xc9pk0xbba9q8WqFfeaY=biLkVcLq=JHqVepeea0=as0db9vqpepesP0xe9Fve9Fve9GapdbaqaaeGacaGaaiaabeqaamqadiabaaGceaqabeaaiiGajaaOcqWF4oqCkmaaBaaajeaObaGaemOAaOMaem4zaCgabeaajaaOcqGG8baFcqWGjbqskmaaBaaajeaObaGaem4zaCgabeaajaaOcqGH9aqpcqaIXaqmcqGGSaalcqqGGaaicqqGYbGCcqqGLbqzcqqGZbWCcqqG0baDcqqGGaaicqGG+bGFcqWGobGtkmaabmaajaaObaGcdaWcaaqcaaAaaiabdweafjabdogaJPWaaSbaaKqaGgaacqWGQbGAaeqaaKaaGkab=D7aOPWaa0baaKqaGgaacqWGQbGAcqWGNbWzcqaIWaamaeaacqaIYaGmaaqcaaQae8hVd0McdaWgaaqcbaAaaiabdQgaQjabdEgaNjabgwSixdqabaaajaaObaGaemyrauKaem4yamMcdaWgaaqcbaAaaiabdQgaQbqabaqcaaQae83TdGMcdaqhaaqcbaAaaiabdQgaQjabdEgaNjabicdaWaqaaiabikdaYaaajaaOcqGHRaWkcqWFdpWCkmaaDaaajeaObaGaemOAaOMaem4zaCgabaGaeGOmaidaaaaajaaOcqGGSaalkmaalaaajaaObaGae83WdmNcdaqhaaqcbaAaaiabdQgaQjabdEgaNbqaaiabikdaYaaajaaOcqWGJbWykmaaBaaajeaObaGaemOAaOgabeaajaaOcqWF3oaAkmaaDaaajeaObaGaemOAaOMaem4zaCMaeGimaadabaGaeGOmaidaaaqcaaAaaiabdweafjabdogaJPWaaSbaaKqaGgaacqWGQbGAaeqaaKaaGkab=D7aOPWaa0baaKqaGgaacqWGQbGAcqWGNbWzcqaIWaamaeaacqaIYaGmaaqcaaQaey4kaSIae83WdmNcdaqhaaqcbaAaaiabdQgaQjabdEgaNbqaaiabikdaYaaaaaaajaaOcaGLOaGaayzkaaGaeiilaWcakeaajaaOcqWF3oaAkmaaDaaajeaObaGaemOAaOMaem4zaCMaeGimaadabaGaeGOmaidaaKaaGkabcYha8jabdMeajPWaaSbaaKqaGgaacqWGNbWzaeqaaKaaGkabg2da9iabigdaXiabcYcaSiabbccaGiabbkhaYjabbwgaLjabbohaZjabbsha0jabc6ha+PWaaSGbaKaaGgaakmaadmaajaaObaGcdaWcaaqcaaAaaiabdogaJPWaaSbaaKqaGgaacqWGQbGAaeqaaKaaGkabdggaHjabdohaZPWaa0baaKqaGgaacqaIXaqmaeaacqaIYaGmaaqcaaQaey4kaSIae8hUdeNcdaqhaaqcbaAaaiabdQgaQjabdEgaNbqaaiabikdaYaaaaKaaGgaacqWGJbWykmaaBaaajeaObaGaemOAaOgabeaakmaabmaajaaObaGaemyyaeMaey4kaSIaeGymaedacaGLOaGaayzkaaaaaaGaay5waiaaw2faaaqaaiab=D8aJPWaa0baaKqaGgaacqWGHbqycqGHRaWkcqaIXaqmaeaacqaIYaGmaaaaaKaaGkabc6caUaaaaa@EC46@

For all iterations, the following are sampled:

cj|rest~[G′bs22+∑g=1G′θjg2ηjg02G′(b+1)]/χG′(b+1)2,p|rest~Beta(∑g=1GIg+1,G−∑g=1GIg+1),Ig~Bernoulli(dg),dg=p(Ig=1|rest)=p Prob(Yg|Ig=1)p Prob(Yg|Ig=1)+(1−p) Prob(Yg|Ig=0).
MathType@MTEF@5@5@+=feaafiart1ev1aaatCvAUfKttLearuWrP9MDH5MBPbIqV92AaeXatLxBI9gBamXvP5wqSXMqHnxAJn0BKvguHDwzZbqegyvzYrwyUfgarqqtubsr4rNCHbGeaGqiA8vkIkVAFgIELiFeLkFeLk=iY=Hhbbf9v8qqaqFr0xc9pk0xbba9q8WqFfeaY=biLkVcLq=JHqVepeea0=as0db9vqpepesP0xe9Fve9Fve9GapdbaqaaeGacaGaaiaabeqaamqadiabaaGceaqabeaajaaOfaqaaeWadaaabaGaem4yamMcdaWgaaqcbaAaaiabdQgaQbqabaqcaaQaeiiFaWNaeeOCaiNaeeyzauMaee4CamNaeeiDaqhabaGaeeOFa4habaGcdaWcgaqcaaAaaOWaamWaaKaaGgaakmaalaaajaaObaGafm4raCKbauaacqWGIbGycqWGZbWCkmaaDaaajeaObaGaeGOmaidabaGaeGOmaidaaKaaGkabgUcaROWaaabCaKaaGgaakmaalaaajaaObaacciGae8hUdeNcdaqhaaqcbaAaaiabdQgaQjabdEgaNbqaaiabikdaYaaaaKaaGgaacqWF3oaAkmaaDaaajeaObaGaemOAaOMaem4zaCMaeGimaadabaGaeGOmaidaaaaaaeaacqWGNbWzcqGH9aqpcqaIXaqmaeaacuWGhbWrgaqbaaqcdaQaeyyeIuoaaKaaGgaacuWGhbWrgaqbaOWaaeWaaKaaGgaacqWGIbGycqGHRaWkcqaIXaqmaiaawIcacaGLPaaaaaaacaGLBbGaayzxaaaabaGae83XdmMcdaqhaaqcbaAaaiqbdEeahzaafaWcdaqadaqcbaAaaiabdkgaIjabgUcaRiabigdaXaGaayjkaiaawMcaaaqaaiabikdaYaaaaaqcaaQaeiilaWcabiqaaqiacaWLjaGaemiCaaNaeiiFaWNaeeOCaiNaeeyzauMaee4CamNaeeiDaqhabaGaeeOFa4habaGaeeOqaiKaeeyzauMaeeiDaqNaeeyyaeMcdaqadaqcaaAaaOWaaabCaKaaGgaacqWGjbqskmaaBaaajeaObaGaem4zaCgabeaaaeaacqqGNbWzcqGH9aqpcqaIXaqmaeaacqqGhbWraKWaGkabggHiLdqcaaQaey4kaSIaeGymaeJaeiilaWIaem4raCKaeyOeI0IcdaaeWbqcaaAaaiabdMeajPWaaSbaaKqaGgaacqWGNbWzaeqaaKaaGkabgUcaRiabigdaXaqcbaAaaiabbEgaNjabg2da9iabigdaXaqaaiabbEeahbqcdaQaeyyeIuoaaKaaGkaawIcacaGLPaaacqGGSaalaeGabaaXciaaxMaacqWGjbqskmaaBaaajeaObaGaem4zaCgabeaaaKaaGgaacqGG+bGFaeaacqqGcbGqcqqGLbqzcqqGYbGCcqqGUbGBcqqGVbWBcqqG1bqDcqqGSbaBcqqGSbaBcqqGPbqAkmaabmaajaaObaGaemizaqMcdaWgaaqcbaAaaiabdEgaNbqabaaajaaOcaGLOaGaayzkaaGaeiilaWcaaaGcbaqcaaQaemizaqMcdaWgaaqcbaAaaiabdEgaNbqabaqcaaQaeyypa0JaemiCaaNcdaqadaqcaaAaaiabdMeajPWaaSbaaKqaGgaacqWGNbWzaeqaaKaaGkabg2da9iabigdaXiabcYha8jabbkhaYjabbwgaLjabbohaZjabbsha0bGaayjkaiaawMcaaiabg2da9OWaaSaaaKaaGgaacqWGWbaCcqqGGaaicqqGqbaucqqGYbGCcqqGVbWBcqqGIbGycqGGOaakieqacqGFzbqwkmaaBaaajeaObaGaem4zaCgabeaajaaOcqGG8baFcqWGjbqskmaaBaaajeaObaGaem4zaCgabeaajaaOcqGH9aqpcqaIXaqmcqGGPaqkaeaacqWGWbaCcqqGGaaicqqGqbaucqqGYbGCcqqGVbWBcqqGIbGycqGGOaakcqGFzbqwkmaaBaaajeaObaGaem4zaCgabeaajaaOcqGG8baFcqWGjbqskmaaBaaajeaObaGaem4zaCgabeaajaaOcqGH9aqpcqaIXaqmcqGGPaqkcqGHRaWkcqGGOaakcqaIXaqmcqGHsislcqWGWbaCcqGGPaqkcqqGGaaicqqGqbaucqqGYbGCcqqGVbWBcqqGIbGycqqGOaakcqGFzbqwkmaaBaaajeaObaGaem4zaCgabeaajaaOcqGG8baFcqWGjbqskmaaBaaajeaObaGaem4zaCgabeaajaaOcqGH9aqpcqaIWaamcqGGPaqkaaGaeiOla4caaaa@28AC@

Here *G *= total number of genes *g*, *G*' = set of genes with *I*_*g *_= 1 in an iteration, and **Y**_*g *_is the data from all studies. Since the full conditional posterior distributions are all closed form when conditioned on the values of *I*_*g*_, the Gibbs sampler [36] is used to generate samples from these distributions. We used 5,000 iterations for all analyses, except for the five study simulation, which required 8,000 iterations, which was more than adequate. The calculations are implemented using the WinBUGS software [44].

## Availability and requirements

The WinBUGS code for executing the models is freely available.

Project name: BayesPoolMicro.

Project home page: 

Operating system: Windows 98 or later.

Other requirements: WinBUGS software version 1.4 or later [44].

License: free.

## Authors' contributions

EMC and JJS contributed to writing the computer code. All authors contributed to the development of the methodology and to writing the manuscript.

## Figures and Tables

**Table 1:** Results for two-study simulations. Integration-driven discovery rate (IDR) and the number of discovered genes for various threshold values of the posterior probability of differential expression, *γ*, and three simulated levels of the percent of differentially expressed genes *p *= 5%, 10%, 25%. The true false discovery rate (*t*FDR) is controlled at 5% for all pooled studies.

**Simulated *p***					
**0.05**	**γ**	0.50	0.90	0.95	0.99
	**IDR**	4.6%	22.1%	33.3%	42.9%
	**# Discovered genes**	109	86	78	70
**0.10**	**γ**	0.50	0.90	0.95	0.99
	**IDR**	3.5%	12.6%	14.3%	29.8%
	**# Discovered genes**	231	191	175	161
**0.25**	**γ**	0.50	0.90	0.95	0.99
	**IDR**	1.9%	3.2%	8.4%	20.3%
	**# Discovered genes**	642	528	499	434

**Table 2:** Number of differentially expressed (D.E.) genes for fixed top numbers of genes. The number of differentially expressed genes discovered by the pooled model and individual models for fixed threshold numbers of top genes, including the two-study simulation model, *p *= 5%, 10%, 25%, and the five-study simulation model, *p *= 10%.

**Two-study simulation data**	**Threshold number of genes**	**# of D.E. genes, pooled model**	**# of D.E. genes, individual study 1**	**# of D.E. genes, individual study 2**			
*p *= 5%	150	128	97	108			
*p *= 10%	300	261	211	218			
*p *= 25%	750	669	569	572			
							
			**# of D.E. genes, individual study number**				
**Five-study simulation data**	**Threshold number of genes**	**# of D.E. genes, pooled model**					
			**1**	**2**	**3**	**4**	**5**

*p *= 10%	300	278	211	218	229	221	216

**Table 3:** Results for *Bacillus subtilis *experimental data. Integration-driven discovery rate (IDR), posterior expected false discovery rate (*pe*FDR) and the number of discovered genes for various threshold values of the posterior probability of differential expression, *γ*, for the pooled analysis of the *B. subtilis *mutant and induction experimental study data.

**γ**	**IDR**	**Posterior expected FDR**	**# Discovered genes**
0.995	53.3%	0.001	89
0.99	50.0%	0.002	96
0.95	28.5%	0.013	130
0.90	20.1%	0.025	144
0.50	8.2%	0.115	194
